# Part I. Hospital Reports

**Published:** 1828-01-01

**Authors:** 


### PRACTICAL MEDICINE;

BEING

### The Spirit of the Medical Journals,

*Foreign and Domestic;*

WITH COMMENTARIES.

---

### PART I.

### HOSPITAL REPORTS.

### 1. INFLUENCE OF THE STOMACH ON THE MIND.*

The intimate sympathy which subsists between the brain and stomach has long been acknowledged; but we are quite convinced that two-thirds of the *effects* of this sympathy fail to be traced to their proper source. Thus, stomach affections are every day attributed to errors in diet, or other common physical causes acting directly on the organs of digestion, when the real cause is a *moral* one, acting through the medium of the mind, or, what is the same thing, through the medium of the mind's organ, the brain. So, on the other hand, we see disordered intellectual functions attempted to be traced to purely *moral* causes, when these causes are purely *physical* ones, deranging the mind through the medium of the stomach. Singular and incredible as the opinion may appear to those who have not much studied the subject, yet we have no hesitation in stating it as our conviction, that—*moral* causes act most frequently on the stomach and other digestive organs, while *physical* causes act most frequently on the mind. This view of the subject would incline one to think that, when a morbid impression is made on any organ—say the brain, as by a piece of bad news or the like, the organ primarily impressed immediately throws the onus on some other with which it sympathises, as, for instance, the stomach or liver. These latter, on the other hand, when disturbed by improper diet, or other causes, shift the suffering from themselves to the brain, in the first instance—thus acting like individuals in this world—every one for himself!

This seems to be pretty nearly the conclusion to which M. Bayle has come. "Every action, (says he) which is somewhat too energetic in

---

* On the Influence of Gastric Affections in the Production of Mental Maladies, and *vice versâ*. By M. Bayle.—*Revüe Medicale.*

one of these two organs (the brain and stomach), awakens a sympathetic affection in the other, and *vice versâ*. It is thus that the work of digestion, particularly if too laborious, or if the food has been in too great quantity, renders the senses more obtuse, exercise less relished, and induces an inclination to sleep. A very small quantity of spirituous potation has this effect on many people who are morbidly sensitive. Tea and coffee, on the contrary, excite the brain and intellectual faculties through the medium of the stomach, so as to prevent sleep for a time—and this before they can possibly be absorbed into the current of the circulation, and thus act directly on the brain." "On the other hand, the influence of the brain on the stomach is not less evident under a great variety of circumstances. The *sight* of nauseous objects often causes sickness of stomach, while that of savoury viands immediately excites an appetite. A blow on the head, an inflammation of the brain, will cause vomitings, and violent pain in the epigastrium."

The main subject of investigation, however, which is here proposed by our author, is, the reciprocal influence of chronic inflammation of the mucous membrane of the stomach and bowels in the production of mental maladies, and of mental maladies in the production of these gastro-enterites. This important investigation is to occupy several Memoirs in the Revue Medicale, and we shall take care to follow M. Bayle as he proceeds. The cases which come forward, on this occasion, will be valuable, whatever may be thought of the doctrine which they are meant to support.

*Case* 1. Madame H——, aged 63, of violent and tyrannical temper, had experienced, for some years, considerable domestic chagrin, without producing any ostensible effect on her mental faculties, till the month of December, 1817, when symptoms of alienation appeared. She became taciturn—was haunted with religious fears—and complained of severe pains in the epigastrium. She consulted a priest, and was evidently worse after receiving spiritual consolation. She was now in constant dread of being poisoned, and refused all aliment—complained of dreadful pains in her stomach—wished ardently for death, and more than once attempted suicide. She could not be prevailed upon to speak on any other subject than that of her hallucination.

In this state, she entered the Maison Royale de Charenton, on the 7th March, 1818. Her complexion was yellow—countenance meagre, and indicative of great melancholy—skin dry and harsh, with a strong tint of yellow—taciturnity obstinate—under constant persuasion that she was going to be poisoned and to undergo unheard of torments; hence evidently the desire of death, the attempts at suicide, the refusal of food, the groans and sighs, and the profound melancholy. This state continued without any alteration except an increase of obstinacy in taking food; so that she was actually destroyed by this obstinacy, and died on the 14th May, in the greatest possible emaciation.

*Dissection*. A considerable serous effusion in the ventricles, at the base of the skull, and in the spinal canal. The arachnoid was rather thickened and opake—the pia mater injected—and the lining membrane of the ventricles thickened, and covered with fine granulations. In the chest, the inferior portion of each lung was hepatized. The heart was sound, but rather flabby and pale. The stomach contained some yellow fluid, and its mucous membrane was throughout reddened, thickened, and, in one place, ulcerated to the extent of two inches in length by one in breadth. This ulcer was in the vicinity of the pylorus—had penetrated through the whole of the coats, and even into the pancreas, to which the stomach was adherent; so that, in fact, the ulcerated cavity in the pancreas made part of the general cavity of the stomach. There were some marks of inflammation in the mucous membrane of the small intestines. The gall-bladder was filled with a calculus, in the shape of a pigeon's egg, to which the parietes of the receptacle were every where adherent.

M. Bayle thinks there is fair reason to believe that, in this case, the gastric affection preceded the mental alienation, and had contributed considerably to it. This female was predisposed to insanity on both father and mother's side; and, therefore, the brain sympathised more readily, and to a greater extent, in this case, with the gastric disease. The author also thinks that the disease of the stomach was exasperated by the mental malady, and by the domestic chagrin to which the unfortunate patient had been exposed. In this we cannot but agree with M. Bayle.

The author throws out a curious hint, or rather opinion, in this place, which seems not entirely devoid of probability—namely, that the *form* of the mental hallucination was determined by the physical malady in the stomach:—That is, that the conviction of being poisoned was occasioned by the inflammation and ulceration going on in the stomach. It is no wonder that, under this impression, and suffering so much from an ulcerated stomach, she should hate, and endeavour to avoid society—refuse food—and attempt suicide! Many a poor wretch has raised his hand against his own life from a disordered stomach, without knowing where the source of his misery lay concealed!

The case above detailed offers another of those thousand ways in which Nature endeavours to combat disease, or at least to resist the consequences of it. The adhesion of the stomach to the pancreas no doubt preserved life, wretched as that life was, for some time; for, had the adhesion not taken place, an extravasation into the abdomen of the gastric contents, would have happened long before.

*Case* 2. Madam G——, an English lady, aged 59 years, of nervous temperament, and sombre disposition, entered the Charenton on the 30th January, 1819, and no precise account of her history could be obtained. It was discovered, however, that she had suffered, for some considerable time, from pain and sense of weight in the epigastrium, and that she had been in possession of her reason till the month of December, 1818, at which time she lost the greater part of her property, and her affairs became sadly deranged. It was then her intellectual faculties gave way, under the moral affliction. She now became possessed with the idea, that her attorney was about to arrest her and all her family, and immure them in prison. She saw and she heard a thousand things to confirm her suspicions, and her whole time was occupied in devising stratagems to elude her pursuers. This hallucination had occasional remissions, during which she partially recognized the baseless fabric of her apprehensions. At other times she had exasperations of the terror above-mentioned, accompanied by violent pain in her stomach, at which periods she refused food, and the bowels were obstinately constipated.

When she came into the CHARENTON, she presented a triste picture of melancholy—paid little attention to what was going on around her—and was evidently occupied entirely with the subject of her hallucination. "She was a lost woman—it was all over with her—she could not long survive, as her own feelings plainly told her—her head and stomach were the seats of dreadful pain—people were endeavouring to poison her." These ideas were so dominant, that she could speak of nothing else. She was in constant agitation—always wanting to get away—refused all aliment—bowels obstinately confined. These symptoms augmented in degree, but never changed their character. At the end of four months, this unhappy patient was in a melancholy situation. Her emaciation was great—she was harrassed with violent cephalalgia, and acute pains in the stomach, sometimes accompanied by a burning heat of skin, and darting pains in various parts of the body. The greatest difficulty was experienced in exhibiting any food, as she was strongly impressed with the persuasion that it was always poisoned. She lingered out till the 28th March, 1820, when long sought death came to her relief.

*Dissection.* There was an effusion of a sero-sanguinolent kind between the membranes of the brain, and in the ventricles. The cerebral substance was softened, but the meninges were not thickened. There was considerable hepatization of the lungs, and three ounces of serum in the pericardium—heart sound. The stomach was remarkably contracted, and took a peculiary lengthened form, from above downwards. The mucous membrane was covered with a thick layer of yellowish mucous matters, and underneath was very red throughout. The liver was gorged with blood, and its right lobe descended to within two inches of the os ilii. The small intestines were very much contracted, and their mucous membrane slightly reddened. The colon was also greatly contracted, and singularly contorted in various directions. All the other abdominal viscera were healthy.

Although the gastric lesions above described cannot fairly be set down as causes of the mental alienation, yet there can be little doubt that the disorder of the stomach greatly assisted the moral causes of the cerebral disturbance. In short, we may conclude that in this, as in a thousand other cases of less serious character, the two organs acted and re-acted on each other—that the physical disorder aided the moral causes in deranging the mind, while the moral causes contributed not a little to the disorder of the stomach.

*Case* 3. M. A. aged 51 years, of nervous temperament, and eccentric character, was the son of a man whose intellects were rather suspected, and had a brother in a state of monomania. For a long time, M. A. complained of pain in his stomach, general discomfort, deranged state of his digestion, &c. which led him to consult a great number of physicians, and swallow an immense quantity of physic. He took a great deal of purgative medicine in particular, and thought he could not live without daily purgation. He became irritable, and quarrelled with most of his friends, though naturally of a good disposition. In 1814, he got into some trouble, in a public office to which he belonged, and was ultimately tried, for continuing an abuse introduced by his predecessors. He was however pardoned by the King in 1815, and in 1818 he got another situation under government. But he now became a violent Bonapartist, and denounced vengeance against the Bourbon race. It was plain that the man was deranged, and, in 1820, he was confined in an asylum. He conceived that all the men of letters in Europe had conspired against him, and that it was impossible he could escape their machinations. He refused food and drink, and emaciated progressively. On the 10th May, 1821, he entered the CHARENTON, in the condition above-mentioned. During the first three months of his sojourn in this establishment, he presented the following phenomena:—Face pallid and sallow—answers pretty correctly to questions put to him, but shews imbecility of intellect—appears calm and tranquil. He conceived a violent friendship for an ideot in the same establishment, and was always at his side, attending to him and imitating his actions. When remonstrated with, he asserted that his friend the ideot was a personage of high rank, and that he himself was commissioned to wait upon him. He was put into another quarter of the asylum, in order to break this attachment. He then devoted himself to constant employment. He was never a moment idle. He complained of pains in his stomach, would eat very little, and began to suspect poison in his food. Emaciation now made great progress, yet still he got out of bed every day, and wandered about the house. On the 2d November, 1821, he suddenly fainted away, and never recovered.

*Dissection.* The arachnoid was slightly thickened, and the pia mater injected. The cerebral substance was softer than natural—no disease in the chest. The stomach was very much contracted, and the internal lining highly inflamed and thickened in its structure, especially towards the pyloric orifice. The mucous membrane of the duodenum was still more intensely reddened. The lining of the upper portion of small intestines was red, which redness gradually decreased as they descended towards the caput coli. The colon was free from disease. The mesenteric glands were enlarged—all the other viscera were healthy.

The same remarks apply to this, as to the former case. The state of the stomach can hardly be considered as a mere coincidence in this case. It was probably an active agent in the production of the mental malady.

*Case* 4. Mad. B. aged 47 years, of nervous temperament, and delicate constitution, had a brother who died in a state of mental alienation. The storms of the revolution had made a deep impression on the young mind of the patient, and she received another shock in 1814, during the occupation of France by the Allies. This shock was not lessened by the fear that her husband would lose his place under government, on account of the part he took after Bonaparte's return from Russia. Towards the end of 1814, she fell into a state of deep melancholy, and now she became haunted with the suspicion of being *poisoned*—of being led to the guillotine, &c. She sought solitude, maintained obstinate silence, refused her food, and attempted suicide. In this condition, with alternate exasperations and mitigations of her complaint, she continued till the year 1821, when she entered the Charenton, on the 6th April. The predominant idea was still that of being poisoned—that there were enemies in pursuit of her, and that her husband and daughter had deserted her. She had such an insurmountable aversion to animal food, that she could not be persuaded to taste it. She lived on a few ounces of bread daily. When food was forced upon her, she said it made her ill, and she frequently vomited it up again. About the middle of May, 1822, she became remarkably debilitated, and began to spit up purulent matter, attended with diarrhœa. She lingered out a wretched existence till the 6th September, when she expired.

*Dissection.* The arachnoid was natural. The pia mater was injected, as was the substance of the brain. The lungs were hepatized in some places, excavated in others, and tuberculated generally. The mucous membrane of the stomach was chronically inflamed throughout, but more intensely so about the pylorus. The same might be said of the small intestines, with the addition of many ulcerations, some of which nearly penetrated through the whole of the coats. The mucous membrane of the colon was reddened, and double its natural thickness. The mesenteric glands were considerably enlarged.

These, and other cases which are to follow, entitle us to conclude, M. Bayle thinks, that the chronic inflammation which takes place during mental alienation tends to keep up the intellectual disorder—to modify its form—and especially to determine that peculiar dread of being poisoned—that aversion to food—and that tendency to suicide, so conspicuous in the above cases.

We shall take up M. Bayle's other memoirs on this interesting subject as they appear in our Parisian contemporary.

### 2. DISEASES OF THE BREASTS.

[St. George's Hospital.]

Nothing can be more important to the practitioner than to be able to distinguish true malignant diseases of the breast, from those morbid growths which appear indeed formidable, but which may be removed with comparative safety and success. An instance of this occurred not long ago at St. George's Hospital.

Ann Dale, æt. 75, was admitted April 18th, 1827, under the care of Mr. Brodie, with disease of the left breast, of which she gave the following account. Six years ago she noticed two small tumours in the axilla; in a few weeks there followed a swelling in the breast, which was attended with very slight pain, but at times a little thin, bloody discharge oozed from the nipple. The swelling proceeded slowly and with little inconvenience, save that occasionally it became red and painful, with slight hæmorrhage from the nipple. She applied to several surgeons, and all concurred in advising an operation. On admission the left breast was as large as a full-grown melon, the enlargement being evidently in the gland itself, and moveable upon the pectoral muscle and ribs, whilst a hard knotty process extended into the axilla, to all appearance an induration of one or more of the lymphatic glands. The tumour was irregular on its surface, having in parts a scirrhous hardness, in parts a doughy, miry feel, not exactly that of fluid; it was extremely heavy—not very painful on pressure—the cutaneous veins were full and tortuous, and in the situation of the nipple a livid looking fungus projected, discharging a little unhealthy pus.

She was affected besides with symptoms of inflammatory action within the chest, but otherwise had always enjoyed robust health, and her appearance was not altogether unfavourable. She had been married, and borne children—the catamenia had ceased long before the commencement of her complaint. By means of bleedings, and salines with antimony, &c. the chest affection was got under, and towards the middle of July she appeared neither unfit nor unwilling for the operation. The breast was accordingly, on the 19th July, removed with great ease. The bleeding was trifling, but wine was given to the patient to encourage it *at the time*, and all the vessels of the least size secured by ligature. The flaps of integument were brought together by sutures and adhesive straps, and pounded ice applied. On examination of the tumour, which weighed three or four pounds, it was found to be, as had been predicted by Mr. Brodie, neither true scirrhus nor fungus hæmatodes, but composed of a yellowish, transparent, jelly-like substance, contained in small cysts or compartments, formed by membranous bands or septa intersecting each other in all directions. In the centre of the tumour was a small cyst containing serum. For the next two days she went on very well, but on the 22*d* there was anxiety of aspect —pulse quick and evidently intermitting—surface hot—tongue inclined to brown. She had had since the operation only one scanty motion. 23*d*. With the aid of the senna draught, in a mixture of haust. salinus, antimonial wine, and tincture of hyosciamus, the bowels have been freely opened this morning. Pulse 126; intermits at every 10th beat—some anxiety and heat of skin. The dressings were removed for the first time, and the greater part of the wound was found to have united; just, however, under the head of the clavicle was a small, and rather painful tumour, apparently formed by coagulated blood. 25*th*. Pulse distinctly intermitting—anxiety—restlessness—some thirst—much pain in the upper part of the breast—tongue brownish. The wound was dressed, and looked not amiss, but the swelling above-mentioned, being very painful, was lanced, and dark, semi-putrid blood, with some highly offensive matter evacuated. 26*th*. Relieved. 27*th*. Not so well to-day, there being another swelling below the line of the wound: it was punctured, and pus, mixed with a little blood, evacuated. From this time, all the bad symptoms rapidly disappeared; whilst, in consequence of opening the two or three small abscesses which had formed, the matter was prevented burrowing, and the adhesions which had taken place were preserved. At present, Aug. 20th, the wound is nearly healed—the cough is trifling—little or no expectoration in the mornings, in short, the good woman looks, and declares she really feels, "many years younger" than before the operation.

*Remarks*. This case shews at what an advanced age these large tumours may be removed from a tolerably healthy patient, with success. The tumour was evidently not true carcinoma; for it was of considerable magnitude, was attended with trifling pain, and had not the stony hardness of cancer, besides which it had lasted for upwards of six years, without having gone on to mischief, which, speaking generally, is not the case with carcinoma. In an excellent clinical lecture* delivered upon the occasion by Mr. Brodie, that distinguished surgeon observed that he had removed several such breasts, and the patients had survived for many years afterwards, without any return of the disease.

In No. 205 of the Lancet, there is, we perceive, a case of fungoid disease of the breast related.

——, æt. 32, of spare habit, light complexion, and unhealthy appearance, was admitted into Guy's Hospital, under Mr. Morgan, with the right mamma enlarged to three times its natural size. The long axis of the tumour was from side to side, it was purplish, especially on the inner side; skin bright—cutaneous veins loaded—large subcutaneous veins ramifying over the tumour. The tumour was tense, harder in some places than others—very heavy, and moveable upon the parts beneath. Axillary glands unaffected. The woman had felt a small lump in the breast for ten years or more, but the enlargement has assumed its present size and appearances within the last fourteen weeks. She had borne several children, and suckled them from this breast. Amputation was performed by Mr. Morgan, on the 28th July. The skin was so far affected, that it could not be saved; the wound was, therefore, dressed in with lint, and left to fill up by granulations. The tumour was inclosed in a dense capsule; it was firm at its base, but, at its circumference, there were small cysts, containing serum.

---

* Mr. Brodie alone, of the Physicians and Surgeons to St. George's Hospital, is in the habit of delivering clinical lectures. Is this as it should be? Are bad examples only *contagious?*

*P.S.* We have just learnt, with great pleasure, that Mr. Rose has caught the infection of giving clinical lectures. We are sure that few can be found better qualified.

We know not whether this may be fairly stiled a case of fungus hæmatodes of the breast, for, in fact, nothing is so difficult as to assign a name and classification to each tumour we come across. Whether it was or was not fungus hæmatodes, Mr. Morgan acted very rightly in removing it, for it bade fair to go on to the destruction of the patient, if let alone. Was the disease originally malignant, or was the "small lump" observed in the breast for ten years previously, a mere chronic enlargement, which, under particular or accidental circumstances, was converted and *ripened*, in so short a time as fourteen weeks, into a large and formidable tumour? The question is more easily asked than answered.

---

### 8. CASES OF INTERNAL STRANGULATION.

[La Charité. M. Louis.]

These cases are uncommon; but they are probably not so much so as is imagined. Modern pathological investigations are daily placing such instances on record.

*Case* 1. A female, aged 34 years, of moderate embonpoint, was admitted into La Charité, on the 15th December, 1824. Her menstrual discharge had been suppressed from the age of 18, and she suffered pains in her loins, and other inconvenience, at stated periods ever since. She had been six days ill before she came to the hospital. The complaint commenced with thirst, loss of appetite, nausea after taking food, shiverings, perspirations at night. Still she had regular, though scanty evacuations from the bowels. On the fifth morning, she had numerous bilious vomitings, with pains in the hypogastrium, which pains became more and more considerable, accompanied by a burning heat there, augmented by pressure. *Sixth day*. The vomiting still continued, and there was obstinate constipation, with an indescribable pain about the anus.

At this period, she was bled from the arm, by a private practitioner, without any relief. On coming into hospital, she presented the following symptoms:—Countenance yellow, and exhibiting signs of prostration—malaise—anxiety—constant restlessness—white but moist tongue—ardent thirst—epigastrium soft, and void of pain—nausea—abdomen rather distended and painful below the umbilicus—constipation—retention of urine—pulse small, quick, and feeble—temperature of skin little elevated. The bladder was emptied by the catheter, and 30 leeches were applied round the umbilicus, to which succeeded fomentations and lavements, but the *latter* could not be thrown up. *16th*. No relief; indeed, the symptoms were rather aggravated, and the abdomen more enlarged. Another relay of leeches—warm bath—castor oil by the mouth. Lavements could not be made to pass up. *17th*. The skin was generally yellow, and the features shrunk, while the abdomen was still more distended, and the constipation as obstinate as ever. M. Chomel examined the rectum with his finger, and found it spasmodically contracted. *18th*. She died.

*Dissection*. We pass over the appearances in the head and chest, as presenting nothing particular. On laying open the abdomen, the small intestines rushed out, and were greatly distended. In several places, they were glued together by coagulable lymph of recent secretion. The strangulation was found in the ileum, 22 inches from the cæcum, and the portion of strangulated gut was two feet in length. The strangulation was effected by means of a ligamentous cord, 21 lines long, and one line broad, which bound the ileum to the sigmoid flexure of the colon. This cord, which was probably slack in a natural state, had formed a kind of noose, through which the knuckle of intestine had unfortunately passed, and thus became strangulated. The more the ileum was distended, the tighter the stricture became. It is difficult to conceive how a band or cord, only 21 lines in length, could thus form a noose, through which so large a portion of bowel had descended; but such was the fact, and, therefore, all the symptoms antecedent to death were easily explained.

*Case* 2. A female, aged 31 years, was admitted into La Charité, on the 30th April, 1827, having been ailing for three months previously. The complaint had commenced with pain of a wandering kind in the abdomen, nausea, bilious vomitings, thirst, and occasional chills, followed by increased heat. These symptoms continued, with more or less intensity, for ten weeks, during which she could not procure alvine evacuations without the aid of lavements. Her appetite had entirely failed, and she lived solely on small quantities of milk. More than a hundred leeches had been applied to the abdomen, without any benefit. For a fortnight before entering the hospital, the constipation had been obstinate, the lavements returning unchanged, and the vomitings were frequent. *May 1st.* Great anxiety—expression of suffering in the countenance—constant change of position—plaintive moanings—the intellectual faculties unaffected. Her tongue was red and dry—thirst urgent—occasional nausea—abdomen voluminous, and, while in pain, the convolutions of the intestines could be plainly distinguished. Leeches were applied to the abdomen. *2d.* The pains were rather mitigated; but no other alteration in the symptoms was perceptible. Purgative lavements were thrown up, and eight grains of calomel were given by the mouth. The patient was in great pain during the remainder of the day, and had two very small motions. She died the next day.

*Dissection.* There was no extravasation in the abdomen, which was principally occupied with the distended convolutions of the small intestines not adherent to each other. A portion of ileum, not far from its termination in the cæcum, was found intimately adherent to the side of the uterus in the pelvis, and there a kind of zig-zag twist or knot had formed, which completely strangled the intestine. The small intestines were, of course, greatly distended with matters above the obstruction, and their mucous membrane was ulcerated in several points. It was observed that the coats of the distended ileum, above the obstruction, were prodigiously thickened, especially the muscular coat—doubtless from the constant efforts which the tube was making to force the obstruction below. This teaches us how the parietes of the heart may acquire a state of hypertrophy, where an obstacle is presented to the flow of blood through the vessel.—*Archives Générales.*

These two cases are the only ones of the kind which M. Louis has observed, during a period of six years, in La Charité, where he examined, in that period, 530 dead bodies. In eight years, we have met with two cases of this kind, in private practice—one of which was that of the late Mr. Belzoni's servant, of which case we stated the particulars in a former Number of this Journal.

---

### 4. EMPYEMA AND PNEUMO-THORAX. BY DR. DUNCAN.

[Royal Infirmary of Edinburgh.]

In the October Number of our northern cotemporary, Dr. Duncan has stated some cases of the above diseases, partly from an interest which he felt " in tracing the progress of his own knowledge," and partly with the hope that they may prove instructive to others. Empyema and pneumo-thorax, Dr. D. observes, " are recognized, during the life-time of the patients, by the common symptoms—or, by the more recent methods of investigating pulmonary diseases, by percussion and the stethoscope. *It is after their presence has been suggested by the* FORMER, *that we have recourse to the* LATTER, *for the purpose of acquiring* CERTAINTY. *These never leave the diagnosis doubtful when they are employed.*" Dr. D. asserts that he was among the first—if not the very first, who made use of the stethoscope and percussion in this country—that he persevered, " notwithstanding the ridicule and sneers of the ignorant and prejudiced." " I have now," says he, " the satisfaction to see that they (auscultation and percussion) are duly appreciated by the *whole profession*—even by those who at first opposed them." Softly, friend Duncan! These means are very far from being duly appreciated by the *whole profession*, or by *one half* of the profession; but they are becoming more and more so every day. What will Dr. Duncan say, when we inform him that, on the day we received his Journal, (2d of October) a professor of physic, and a public lecturer in this metropolis—one, too, who is not more than 40 years of age, and, consequently, not past the period of improvement—publicly denounced the stethoscope as a French bauble, or piece of quackery, which he would never countenance! There are many of the most eminent physicians and surgeons in this metropolis who entertain the same sentiments as the professor above-mentioned; but they are beginning to be less clamorous against the study of auscultation, because they have just discrimination enough to see which way the cat jumps. Nay, there are many who *pretend* to a knowledge of an instrument which they deeply hate and secretly curse, because the study of it interrupts the placid course of their routine practice. We saw one of these worthies the other day put on a look of great wisdom—apply the wrong end of the stethoscope to his ear—hold the other end about two inches from the patient's chest—and sagely remark, that he heard, very distinctly, the respiratory murmur! This respiratory murmur was no other than the murmur of a hackney-coach, passing over a piece of newly macadamized street, opposite to the patient's house! Were it not that the subject is very serious, we could relate some anecdotes of great physicians, which would be very likely to create a smile in the most saturnine countenance. We shall venture to state one instance which occurred to us this very day—2d of October, in the year 1827.

Mr. Charters, an eminent coach-builder in this metropolis, who has consulted almost every physician and surgeon, of any reputation, both in this country and the Continent, presented himself to us—observing, with a significant and intelligent smile, that he came more from curiosity, than with any hope of advantage. We were determined to return the compliment, though we took care that he should not see the drift of our particular enquiries. By a little management, we induced the patient to return home, and collect the prescriptions and written opinions which he had received from the lions of physic and surgery. These he submitted to us—not without an air which sufficiently indicated the *degree of respect* in which he held medical opinions generally. God forbid that we should be so base as to turn any of the documents which were placed in our hands to the detriment of any individual. But we will say, that a statement of this gentleman's case, and a detail of the opinions and prescriptions which he had received, would form an appendix to "Wadd's Mems. Maxims, and Memoirs," which might rival the whole collection which that indefatigable surgeon has compiled! The venerable Portal, now on the verge of 80, is in no danger of suffering from, or even knowing, any criticism which we make on his opinion. This opinion occupies nearly two foolscap pages, and sets out with stating, that Mr. C.'s liver is so enlarged as to reach nearly to the pubes, on the right side. It then descants on the physiological effects of this enormous liver on the organs and functions of the thorax and abdomen, concluding with very minute directions as to diet and medicines, with the view of counteracting these derangements of function in the contiguous viscera. After a most accurate examination of the patient, in the horizontal and vertical positions, we declare that no part or portion of the liver descends below the ribs, or can at all be felt, even when Mr. C. holds in his breath and strains downwards! The patient is so thin, that the aorta can be distinctly traced, from the origin of the cœliac artery to its bifurcation—and in such a person, it is needless to say that an enlarged liver could not possibly be overlooked. Now it appears quite evident, that M. Portal fell into some long reverie or dream of early life, and unconsciously portrayed, on his large foolscap pages, the case of some patient who had passed under his hands half a century previously! If any thing could induce men, when entering their dotage, to reflect on the lot of humanity, and retire before their loss of intellect becomes conspicuous to the world, the above instance might prove a useful hint. We shall draw a veil over the incongruous opinions and wild plans of treatment which are scattered on the record preserved by Mr. C. To our apprehension, the opinion of Magendie came the nearest to truth:—namely, that there was dilatation of the right chambers of the heart, and some degree of hepatization of the lungs. As there is scarcely a medical man of fame in this metropolis who must not recollect the case of Mr. C. the above anecdotes may be interesting to them.

We shall now notice one or two of the many cases which Dr. Duncan has put on record in the last Ed. Journal.

*Case* 1. Mark Young, aged 33, had been under Dr. Home, in the clinical ward, when Dr. D. took charge of it, on the 1st Feb. 1815. Dr. H. treated the patient for hydrothorax. For some time, he had laboured under such painful micturition as to mask the thoracic affection. His whole complaint was of the pain in the urethra, before and after making water, to which the calls were very frequent. He had also difficulty of breathing in going up an ascent—fluttering at the region of the heart—pain on pressure at the epigastrium, and also in the hypogastrium. The difficulty of breathing came on five years previously, after an inflammatory attack. Dr. Duncan's whole attention was directed to the disease of the urinary organs, but entirely failed in affording the patient any relief. It is stated, however, that he had cough and difficult expectoration, with a pulse at 146, small and sharp. In this condition the poor fellow expired, being completely worn out. The dissection was performed by the late Dr. Gordon.

*Autopsia.* The pleura costalis of the *right side* was observed to be much thickened, and, in that side, there were 130 ounces of opake fluid, resembling the serum of the blood, but which, on being agitated, looked like cream. There were flakes of coagulable lymph in various parts of this side. The lung was compressed to about one eighth of its natural size, but seemed otherwise healthy. There was a small abscess in its upper part, and numerous small abscesses were dispersed through the substance of the other lung. There were three ounces of clear fluid in the pericardium, but the heart itself was healthy. The left kidney and ureter were free from disease; but the projecting papillæ of the right kidney were in an ulcerated state, and their membranous coverings, the infundibula, nearly destroyed. The pelvis of this kidney was enlarged, and filled with a granular matter, resembling particles of inspissated pus. The ureter was much dilated. The inner coat of the bladder was inflamed throughout, and, in many places, ulcerated. The prostate gland and urethra appeared free from disease.

Dr. Duncan has little doubt that this man's death is to be ascribed solely to the disease of the urinary organs—and "that his fever and emaciation proceeded entirely from irritation and want of sleep." We cannot agree with the learned Professor on this point. Such a collection of purulent matter in the right side of the chest, with a diminution of that lung to one eighth its natural size, while the other lung was universally studded with small abscesses, and the bronchia filled with pus, were circumstances quite sufficient to destroy life—and cannot be left out of the cause of death, notwithstanding the inflammation and ulceration of the bladder.

We were a little surprised to find that Dr. Duncan, who takes the credit of first practising *percussion* in this country, should have entirely overlooked this diagnostic measure in the above case, so late as 1815—long after we ourselves had drawn the attention of the profession to "*thoracic percussion and abdominal pressure*," in formal papers on the subject.* A very superficial examination of the chest would have detected the state of things in the above case.

*Case 2.* Rebecca Neilson, aged 25 years, complained (12th Nov. 1820) of oppression and difficulty of breathing—frequent cough—purulent expectoration—palpitation at the epigastrium and beneath the *right* mamma;—all which symptoms were increased by the slightest attempt at motion. She could only lie on the right side, and could not take in a full inspiration. The right side of the thorax was very sonorous, and the respiration was puerile there. The left side was dull, and there was an obscure sense of fluctuation. The entrance of the air into the air-cells was indistinctly heard in all parts of this side. The action of the heart was faintly heard in the præcordial region, and very evidently on the right side of the sternum. There was a small tumour, conveying an emphysematous feel, in the left side of the chest, where she had lately received a blow. Her complaint had been of some months' standing, and had followed sleeping in a damp bed. She died on the 24th of November, 12 days after she came under Dr. Duncan's care.

*Dissection.* There were four pints of purulent matter in the left side of the chest, by which the heart was pushed over to the right of the sternum. The pleura was somewhat diseased, and covered with false membranes. The left lung was not larger than a common-sized spleen, and there was no communication between the interior of this lung, and the general cavity of the pleura at first discovered; but, after a more careful examination, an opening, the size of a goosequill, was found, terminating in a pretty large bronchial tube. The lung was partly sound, and partly hepatized. There were a few ounces of serum in the pericardium.

Dr. Duncan has no doubt that there was originally a mixture of air with the fluid, but that it was absorbed. This is very probable.

*Case 3.* This was one of simple pneumo-thorax. The patient was a young man, who became phthisical after hæmoptysis. At first he could only lie on the right side, and was threatened with suffocation if he turned on the left. "Suddenly, after his expectoration had been unusually copious, he could only lie on the left side, and, instead of having the placid tranquillity of a phthisical patient, he became affected with excruciating pains, which he described as if his inside were tearing out, and referred particularly to the lumbar region." Death soon put a period to his sufferings. On percussion of the corpse, the right side was

* See Medico-Chirurgical Journal: see, also, a translation of Desault's Memoir on this subject, in the 10th volume of the New Medical and Physical Journal, for 1815.—*Ed.*

much more sonorous than the left, which led our author to conclude that the right lung was more healthy than the other. On opening the body, however, the right side of the chest was found filled with air, and the lung compressed into a small space against the mediastinum. There was no communication between the air-tubes and the cavity of the chest. Dr. Duncan thinks that this was originally a case of empyema—and that, so long as the patient could lie only on his left side, the right was filled with purulent matter, which, in progress of time, had found its way into the bronchia,* and been discharged by the mouth, while air entering through the same passage into the cavity, changed the disease into pneumo-thorax.

*Case* 4. Margaret Mac Cromby, aged 32, was admitted the 13th July, 1827, having great dyspnœa, obliging her to lie with the head much raised—decubitus dorsalis—respiration much accelerated, 50 in the minute—left ribs little elevated during inspiration—cough—scanty and difficult expectoration—pain in the left side of the chest—palpitation—profuse nocturnal perspirations—severe diarrhœa—pulse 136—great thirst. The sound, on percussion, is very obscure over the whole left side of the chest, except beneath the clavicle, where it is preternaturally clear and sonorous. On the right side it is natural. No respiratory murmur can be heard in any part of the left side, except in a small space between the spine and scapula, where it is *bronchial*. The *tintement metallique* is distinctly audible on any sudden change of position. On the right side, the respiratory murmur is distinct in the superior lobe, and obscure in the lower portion. The heart is much displaced, its pulsations being most strong near the right mamma. On succussion of the trunk there is evident fluctuation.

After a difficult parturition, 15 months ago, she had considerable hæmoptysis, with much cough, which was relieved by venesection. During the Summer, she continued pretty free from pectoral complaints; but they returned in the Winter. Six weeks before the date of report, she was seized with rigors, acute pain in the left side of the chest, urgent dyspnœa, and increase of cough. These symptoms rapidly increased, with perspirations and progressive emaciation. She died on the 18th July.

*Dissection.* The left side was filled with air and purulent matter, the latter amounting to 8 pints. The heart was pushed to the right side, beyond the median line. Two fistulous communications between the bronchia and the cavity of the pleura were detected by insufflation through the trachea. The lung on that side was greatly compressed and condensed, sinking in water. There was considerable serous congestion of the right lung, which was soft and pulpy.

For several other interesting cases of empyema and pneumo-thorax,

---

* We are glad to see that we are supported by the learned professor in using bronchia in the plural number. We think bronchium and bronchia better than bronchus and bronchi.

we must refer to the Journal already quoted. It gives us much pleasure to see that the study of auscultation and percussion are steadily—indeed rapidly advancing; and we think we are justified in asserting, that this Journal has been very conducive to this important improvement in medical science. We were the first to give an extended analysis of Laennec's work,* and we have never ceased, during the last eight years, to enforce the value and the utility of the study of auscultation.

---

### 5. DISSECTION OF AN EPILEPTIC.

[Bicêtre.]

The following case is published by M. Bosc, of the Bicêtre. A young man, named Lecoq, aged 17 years, an epileptic, was received in the above Institution in the month of December, 1826, and placed in a surgical ward, on account of a caries of the phalanges of one of his fingers. He was of feeble constitution, his limbs but little developed, and his flesh flabby and emaciated. His intellectual powers were almost annihilated, if they did ever exist. He made use of no words, except the monosyllables *Yes* and *No.* The epileptic fits were not frequent. He always slept on his right side in bed, with his head under the coverlet. He complained of no pain, but had a diarrhœa upon him during the three months before he died in the hospital. This complaint resisted all medicines, and was supposed to depend on ulceration of the intestines. The abdomen was extremely retracted. He wasted away gradually, and, at length, died.

*Dissection.* The cranium presented a sugar-loaf form. The meninges were sound. There were several depressions on the surface of the hemispheres corresponding to protuberances of the skull; and, in these depressions, as well as in several other parts of the brain, the cortical substance was soft, and almost diffluent. The anterior lobes of the brain were extremely little developed—the circumvolutions small and numerous—the anfractuosities shallow. It was found that the general surface of the brain was softened, as far as the cortical substance was concerned; but, below this, the medullary matter was so indurated as to resemble the white of a hard-boiled egg. This extreme degree of induration was particularly observed over the lateral ventricles. The bottom of the same cavities presented considerable softening of the medullary substance. There was some serum in the ventricles. The cerebellum appeared sound, but was remarkably small. The spinal marrow was not examined. The contents of the thorax were natural. In the abdomen, the peritoneum was found to be the seat of extensive chronic inflammation, the convolutions of intestines being glued together by false membranes. There was a plentiful crop of miliary tubercles developed under the peritoneal covering. The mesenteric glands were in a state of disease. The intestines themselves appeared sound:—their mucous

---

* See Medico-Chirurgical Journal, for January, 1820.

membrane was pale, and somewhat softened; and the mucous follicles were considerably developed in the colon and rectum, but unattended with any ulceration. There was no other change of structure discernible in any of the abdominal viscera, except some equivocal traces of disease in the liver.

The state of the brain sufficiently accounts for the condition of the intellectual faculties, and, on this, doubtless, were dependent the epileptic paroxysms. The state of the peritoneum proves, that chronic peritonitis may go on a long time without shewing any very prominent symptom of its existence. The diarrhœa, which continued so long, and which, indeed, appears to have carried the patient off at last, was evidently not the result of inflammation of the mucous membrane of the intestines, to which it is usually referred. It is more probable that, in this case, as well as in many others, it was connected with derangement of the biliary secretion.—*Bibliotheque Med.*

---

### 6. EMPYEMA. BY M. GASC.

[Hôpital de la Garde Royale.]

A soldier, 29 years of age, of robust constitution, was exposed, in the beginning of January, 1827, to severe cold in the night, as well as fatigue, and had the imprudence to quench his thirst with half-melted snow at the time. Next day, he was seized with pain in the left side of the chest, low down, accompanied by sense of oppression, cough, and some expectoration of a bloody quality. He did not apply for medical assistance, and remained several days without any. He remained more or less ill till the end of February, sometimes keeping his bed—sometimes doing duty. On the 1st of March, the symptoms of pleuro-pneumonia became so intense, that he was conveyed to the Hospital of the Guard, on the 4th of the same month. When examined, on the 5th, he was found to have acute pain in the left side of the thorax, below the nipple—laborious respiration—cough and sanguinolent expectoration—inability to lie on the right side. Percussion elicited a dull sound in the lower part of the left side—no respiratory murmur could be heard there by the stethoscope—and the "râle crepitant" was heard about the nipple. The upper part of the lung, on that side, was ascertained to be sound. The pulse was hard, quick, and full—heat and pain at the epigastrium augmented by pressure—tension of abdomen—constipation—thirst, anorexia, white tongue—dry skin—interrupted sleep—cough, and embarrassment in the breathing. The diet was reduced low—and sixteen ounces of blood were taken from the arm, which was repeated on the 6th. On the 7th, 30 leeches were applied to the chest. On the 8th, the symptoms were all greatly ameliorated—and this amelioration continued during the 9th; but, on the 10th, they all returned as bad as before; the oppression being great, and the sanguinolent expectoration copious. He was bled again to 16 ounces, and leeches were applied to the lower part of the sternum. The patient went on, sometimes better, sometimes worse, till the 14th, when the symptoms of thoracic effusion were very evident. The action of the heart was distinctly felt in the right side of the thorax—the left side was bulged out—fluctuation was evident, both to the patient and the medical attendants—pulse intermittent.

It was now clear that nothing but the operation could afford any mitigation of the patient's sufferings, and Baron Larrey was the operator. The Baron made an incision between the second and third false ribs, equi-distant from the sternum and spine, and, cutting close along the superior edge of the third rib, gave vent to about 15 pints of sero-sanguinolent fluid. Air then began to enter the thorax, and the wound was closed. During this operation, the viscera, both of the thorax and abdomen, which had been pushed from their natural situations by the large collection of fluid, were restored both to their site and function. The heart pulsated against the canula in the wound. The patient had some hours repose, but, towards the evening, fever became lighted up, and the oppression of breathing as bad as ever. The catheter was again introduced, and some more pints of fluid were drawn off. In the night, the patient suffered much, and fresh symptoms of effusion appeared. An elastic catheter drew off eight more pints of fluid. On the 3d day after the operation, the patient died.

*Dissection.* The pleura of the left side of the chest was completely disorganized, being of a deep brown colour, and, in some places, three or four lines in thickness. There were, also, layers of coagulable lymph, and many fragments of false membranes floating in the remaining fluid. The lower portion of lung, on this side, was disorganized, and completely imbibed with the same kind of fluid which was found in the sac of the pleura. There were about sixteen ounces of limpid fluid in the pericardium. The heart was small and flabby. There were some traces of inflammation in the abdomen; but no disease worth relating. —*Journ. Géněral de Medecine.*

*Remarks.* Although, on the principle of Euthanasia, it is just and proper, that issue should be given to collections of matter, or water, in the bags of the pleura; yet, we need seldom hope for permanent success, where the inflammation producing the effusion has continued long. There is generally so much disorganization in the lining membranes of the chest, and even in the lungs or heart, that little chance of recovery remains. It is somewhat more hopeful when abscess of the lung has burst into the chest, and an opening is soon given for the extravasation. In such cases, recovery is not exceedingly rare.

### 7. PULMONARY ABSCESS.

[Dr. Chambers—St. George's Hospital.]

One object of Dr. Chambers' paper is to show the very rare occurrence of suppuration of the lungs consequent on common inflammation. This last-mentioned process usually goes the length of causing an adhesive deposit in the pulmonary structure, not intense enough to induce suppuration, but sufficient to produce induration, or, as it is more commonly called, HEPATIZATION. In 15 years' observation at St. George's Hospital, Dr. C. has only met with three fatal cases of pure phlegmonous abscess of the lungs, out of nearly 600 dissections of pulmonary disease. Three or four cases have been cured in that period. One favourable and one fatal case of this rare disease are then related. Of these we shall take some short notice.

*Case* 1. A labourer, aged 34 years, was admitted on the 5th January, 1825, much emaciated, complaining of difficulty of breathing—heavy pain in the left side—troublesome cough—copious expectoration of a brownish colour and very fetid smell, with some admixture of blood. He cannot lie on either side, on account of his cough. He has no regular hectic fever, but occasional shiverings, flushings, and perspiration. The pulse was 130, and small—skin cool—tongue of a livid colour, smooth, and clean—bowels open, &c. He stated that some months previously he had received a blow on his left side, ever since which he had had more or less pain in that part; but within the last month this pain had greatly increased, accompanied by the symptoms above detailed. He was put on milk diet, and to take saline draughts with acacia, digitalis, and vinum ipecacuanhæ. Extract of colocynth and lettuce were given at night. Under this treatment the patient daily improved till the 21st January, when the mistura ferri comp. was given, with a pint of porter daily. By the 14th March he had gained flesh—the cough was diminished—and all the functions nearly natural. He was now made an out-patient, and ultimately recovered.

Although there is very little proof in this case of pulmonary abscess, since all the above-mentioned phenomena often result from bronchitis; yet we have no inclination to question the accuracy of Dr. Chambers' diagnosis. Dr. C. speaks most respectfully of Laennec in the beginning of the paper; and yet not the slightest attention is paid to auscultation or percussion in the examination of the patient! The fetid nature of the expectoration seems to form the sole basis of the doctor's discrimination. Well! Be it so. Dr. Chambers is not the only one who scorns any thoracic examination of the mechanical kind. While this paper was before us, a man presented himself with a sheaf of prescriptions from one of the very first physicians (in point of extent of practice) in this metropolis. They were almost all composed of guaiacum and the old farrago of medicines prescribed for *rheumatism*, which was pronounced to be the disease under which the poor man (a gardener at Pentonville) had laboured for two years—always getting worse. On stripping the thorax, the upper portion of the right side, under the clavicle, was bulged out, and the most common examination shewed an aneurismal state of the ascending aorta and innominata. The pulse at the right wrist was annihilated by the pressure of the aneurism on the subclavian. There was pain all along that arm, and across the chest, with inability to lie down in bed. And this was the *rheumatism* for which he was, for more than a year, treated by a physician who has received, at least, five hundred thousand pounds from the public—and whose fiat is fate! Now this same physician is a very good physician—and had he been present when the man's thorax was bared, he would have detected the disease in one moment. But even this trouble will not be taken by any medical man, above the age of 45, in this metropolis—although the above age is that at which fools are said to be converted into physicians, without any study.

*Case* 2. J. Hayward, ætat. 45, admitted October 4th, 1826, much emaciated, complaining of difficult breathing, cough, expectoration of foul and highly offensive purulent matter, of a dark yellow colour, partly tinged with blood. Breath is very fetid—pain in both sides of the chest, aggravated by any attempt to breathe deeply. Lies on his back—pulse 110, small, and sharp—skin hot—tongue red at the sides and furred in the middle—bowels open—appetite indifferent. Says he was attacked eleven weeks ago, with symptoms of inflammation of the lungs, for which he was not bled. The symptoms subsided in a fortnight, and were followed by the above-mentioned expectoration, and the other symptoms already noticed. He was placed on fever diet—to lose eight ounces of blood—and to take saline medicines with antimony, &c. The blood was inflamed—pain relieved—pulse lowered to 80—and the cough rendered less troublesome. *Rep. medicament.* In this state he remained till the 8th of October, when a bowel-complaint came on, which was relieved by small doses of Dover's powder; but on the 14th his strength seemed, all at once, to fail him, and wine and brandy were allowed. He died the next day.

*Dissection.* The lower lobe of the left lung was found to be hepatized posteriorly—in the right side were a few ounces of opake serum. The apex of the left lung was enveloped in thickened pleura, and when this lung was cut into it disclosed two cavities, each the size of a small orange—one in the upper, one in the middle lobe. The first cavity was lined with a loose sloughy substance, and filled with dark yellow fetid matter. The second was empty, the pus having been expectorated through the bronchial tubes, which were seen communicating with it. This cavity also was lined with a very thin membrane of a dark colour. The lung in the neighbourhood of the abscess was rather condensed, but there was no appearance of tuberculation in either of the lungs.

These two cases are considered by this very intelligent physician, as fair specimens of the disease under discussion. In the absence of precise information of the early symptoms (which can seldom be obtained from the uneducated classes) Dr. C. thinks that the phenomena above described will "be generally decisive of its real character." The appearance and smell of the expectoration "are totally different from those which belong to the sputa of tuberculous consumption." "In apostema of the lungs, the expectoration is of a brownish or greenish-yellow colour, and has *an intense odour of putrefaction.*" "This colour was correctly compared, in my hearing, by a physician of considerable experience, particularly in diseases of the lungs, to that of *rotten eggs;*

an appearance in the expectoration, which, he said, he had long been accustomed to consider, when joined with fetor, as holding out a more favourable prospect of recovery to the patient, than that of ordinary purulent matter." This appearance is doubtless owing to the admixture of pus and blood with the particles of sloughy lung which form the parietes of the abscess. In those cases which terminate favourably, the dark colour of the expectoration disappears, and its fetor gradually decreases. The difference between this kind of sputa, and the white or yellow, and inodorous expectoration of tubercular phthisis, is obvious enough. The next distinctive mark, is the absence of hectic fever, after the abscess is fairly opened into the air-tubes. There is also a want of that clear complexion and bright colour so generally attendant on real phthisis. On the contrary, there is a dull muddy sallowness. At the same time, Dr. Chambers does not mean to say that these distinctions are clearly marked and infallible in all cases. There are exceptions here as well as in other diseases. Still the foregoing distinctions may serve to guide our prognosis.

In respect to the treatment, it will be seen, by the cases above detailed, that the separation of the sphacelated part of the lung, of which the parietes of the abscess are formed, is often accompanied, even in an advanced period of the complaint, with attacks of inflammatory action, requiring depletory measures. As soon, however, as this stage has passed over, the patient will often be found to thrive and fatten under the administration of chalybeates and other tonics, with nutritious diet—means which may be resorted to, even when the cough is troublesome, and the expectoration purulent and bloody.

Before closing his paper, Dr. Chambers very modestly ventures on a little bit of theory, to which, indeed, the best constructed minds are occasionally disposed. It is on the long disputed *mode of formation* in tubercles of the lungs. His impression is, that the original nuclei of these tubercles are formed in the mucous glands or follicles of the membrane lining the extreme air-cells and air-tubes—" these glands or follicles being choked up with their own coagulated secretions, and, often coalescing with each other, become, as I conceive, the source of irritation, and subsequent ulceration, of the surrounding tissue." This theory is probably as good as any other that has yet been formed, not even excepting the hydatid theory of Dr. Baron. But we should be glad to see how Dr. Chambers will apply this doctrine to tuberculation of the *serous* membranes, and of the medullary or cortical substance of the brain—states of disease almost as common as tubercles in the *mucous* follicles of the lungs. Dr. C. promises to return to this subject, and we throw out the above hint, merely to afford his ingenuity an additional field for the acquisition of laurels. " We conquer difficulties by daring to oppose them." There are few physicians possessed of more excellent judgment than Dr. Chambers; but medical theory is an enterprize of such a hazardous and arduous nature, that we tremble for our bravest and wisest friends, when we see them fairly embarked in it. To none do we wish more complete success than to Dr. Chambers.—*Med. and Phys Journal.*

### 8. HOSPITAL REPORT FROM THE VAL DE GRACE. BY M. BROUSSAIS.

[For November, December, January, February, and March, 1826-7.]

M. Broussais's doctrine has lately been assailed, through the medium of the practice which it inculcates. It has been said that the success of the Professor in the Val de Grace has not been equal to that of other physicians entertaining different views from the founder of the new doctrine. It is hardly fair to judge of a doctrine or practice by comparative success in different hospitals—or even in the same hospital at different times, or under different physicians. A man may have a run of bad or good luck in the reception of patients, as well as in throwing dice or playing at cards—and this may give a very pleasing or gloomy cast to the numerical results at the end of the quarter or half-year. M. Broussais has caused his aide-major (M. Cassimir Broussais) to present a semestral report from the Val de Grace, appealing, for the authenticity and truth of the report, to the records of the institution, and the evidence of those who walked the hospital at the time. Nothing can be more unexceptionable than this plan, with the reservations above alluded to—and we shall now proceed to give an analysis of this report.

It is asserted by the reporter, that when M. Broussais takes his turn of duty in the Val de Grace, he desires that the worst cases may be sent to his wards. This is magnanimous—more so than wise, perhaps. In the five months above specified, there were entered 438 patients, of whom 20 died, or about one in twenty-two. This certainly is not a very great mortality, considering that an epidemic raged during part of the time, before which, the mortality was only one in thirty-five—and that 86 cases remained in hospital from the preceding semestre. M. Cassimir asserts, (and there can be no reason to disbelieve him) that many were sent to the Val de Grace, in the above period, merely to die. This happens in all hospitals, and must ever prevent a fair estimate of medical treatment. We do not deem it necessary to give the whole table of maladies. Suffice it to say, that there were three aneurisms of the heart—76 cases of acute bronchitis—8 of colitis—7 of duodenitis—3 of encephalitis—60 of acute gastro-enteritis—15 of hypertrophy of the heart—35 of intermittent irritation (ague)—4 of laryngitis—79 of acute pleuritis—29 of other acute inflammations of the thorax.

1. *Pleuritis.* Of the 79 cases of acute pleuritis, only one proved fatal, and that from purulent effusion into the cavities of the pleura and pericardium. This inflammation had commenced five days before the patient's entrance into hospital. In all these cases of pleuritis, the disease was combated by the application of leeches to the pained part. In 29 cases venesection preceded leeching. In general, a single application of 15, 20, or 30 leeches was sufficient. In three cases only was it necessary to have recourse a third time to leeching. Emollient cataplasms always succeeded the leeches, and diluent mucilaginous drink was plentifully given. In six cases, it was necessary to blister after leeching, and, in four of these, the measure was successful. It was remarkable that, in most of these cases, the pulse fell immediately the blisters had risen. In two cases, however, they were applied too soon, and the râle muqueux and fever obliged M. Broussais to have recourse to more leeches. In the great majority of cases, bronchitis preceded the pleurisy, which induced M. Broussais to suppose that the inflammation of the mucous membrane, having arrived at the ultimate ramifications of the bronchia, passed on to the serous membrane, and then produced the corresponding phenomena. When the bronchitis persisted, which was generally the case, leeches were applied under the clavicles, at the top of the sternum, and wherever the râle muqueux could be heard.

These thoracic inflammations were far from being uncomplicated. In 20 cases, at least, there was considerable gastric irritation, which yielded, however, to leeching the epigastrium. In two cases there was evident duodenitis—and, in three instances, the inflammation spread to the other intestines, producing diarrhœa—and to the brain, giving rise to delirium. These cases are detailed at length, but we pass them over.

There were very few instances of relapse in these pleuritic cases. The medium period of residence in hospital was 23 days. It is remarked, however, that M. Broussais never permits a soldier to leave the hospital till he is so completely recovered as to enter immediately on his military duties.

2. *Acute Bronchitis.* Of 76 cases of this disease, M. Broussais lost one. General and local bleeding, especially the latter, was principally trusted to—the leeches being applied to the places mentioned above. There were seldom more than 20 leeches applied at first—and afterwards a small number were applied wherever the râle could be distinctly heard. Blisters were employed in only seven cases. In about 20 cases of bronchitis the inflammation spread to the mucous membrane of the stomach, requiring leeches to the epigastrium. It was surprising to see how soon the detraction of blood from this quarter calmed the irritation of the whole system, and reduced the fever. In many of these cases the appetite came on quickly after the leeches, and it was difficult to restrain the patients from committing excesses. More relapses, however, were occasioned by exposure to atmospherical vicissitudes than by imprudence in diet. The mean term of residence in hospital for this inflammation was 14 days. One case proved fatal. The young man had had cough during the whole of the winter, and was affected with acute bronchitis fifteen days before he was sent to the hospital. He was then spitting up large quantities of purulent matter, and was unable to lie down in bed. He died on the fifth day after he was received into hospital. The trachea and bronchia were found filled with muco-purulent matters, and the lining membrane intensely reddened. The parenchyma of the lungs was, in some places, hepatized. The mucous membrane of the stomach, and also of the jejunum, was inflamed. The patient, therefore, evidently died of suffocation from the effusion into the air-passages.

3. *Pneumonia Acute.* Of sixteen cases of this disease, three died, and a fourth remained doubtful. They were all accompanied by great congestion of blood, not only in the chest, but in the abdomen and other parts, rendering the treatment very difficult. One, two, or three general bleedings were followed by leeches to the chest, or to whatever part appeared to be the seat of congestion. In six cases only were blisters applied. Diminution of the force and frequency of the pulse—of the râle crepitant—of the dull sound—of redness on the cheeks;—and, on the other hand, the facility of expectoration were the signs for discontinuing depletion, and trusting to the efforts of Nature. If, after these favourable phenomena appeared, there was heard any râle in any part of the chest, then a blister was applied. Mean stay in hospital for pneumonia was 22 days.

The first of the three fatal cases died on the fifth day after he was received into hospital, having been ill for twelve days previously. The depletive system was pursued as far as was consistent with prudence, but it was too late. On dissection, considerable portions of lung were found hepatized, and much muco-purulent matter could be squeezed from the rest. The brain was sound; but the mucous membrane of the stomach was highly inflamed, and there were ulcerations in the ileum.

The second patient, whose case proved fatal, had been ill only four days, according to his own account. When received, the dyspnœa was great, and he was spitting up bloody expectoration, with hard full pulse, great heat of skin, and ardent thirst. One general and one local bleeding somewhat relieved these symptoms; but the inflammation spread to the digestive apparatus, and required many leechings. The patient appeared to be convalescing, when a relapse took place, and then all means failed. On dissection, the posterior half of the left lung was found hepatized, and a considerable portion of the other lung was in the same condition. The mucous membrane of the stomach was softened, and there were marks of inflammation in the mucous membrane of the small intestines.

Before taking up the subject of chronic inflammation of the lungs, M. Broussais thinks it necessary to say a few words respecting those acute thoracic inflammations which were on the point of changing into chronic, and which would have certainly induced phthisis, had it not been for the rigid antiphlogistic means that were used. Of 200 patients that entered the hospital during five months, and who were affected with pulmonary inflammation, only one has died of phthisis. In eleven cases, however, the inflammation proved obstinate, and phthisis was *menaced.* The following are the signs, M. B. observes, which indicate that chronic inflammation is taking place, to end in pulmonary phthisis. When patients, relieved from the acute symptoms, and especially from those of gastric irritation, begin to recover their appetite—become cheerful—and regain some degree of strength. They think themselves well, in fact, and regard the remaining cough as nothing. But the attentive physician will readily perceive that, notwithstanding these appearances of amelioration, a focus of inflammation remains. There will be found some râle muqueux or râle sibilant, or both—the sound will be less clear, on percussion, over these points of the chest—the breathing will not be quite free—the chest will be raised, en masse, on inspiration; or one side will rise more than the other—the cough still continues, though much diminished—there is expectoration of a yellow mucus, or muco-purulent fluid—the pulse is more frequent than natural, and more full, especially towards evening—there is some pain or uneasiness complained of, under the sternum, at the epigastrium, or in the throat—the skin is dry and hot in the day, and often covered with perspiration in the night—the features of the countenance indicate some internal suffering, however the patient may endeavour to conceal it, which he almost always does. When patients are examined by the stethoscope they will breathe remarkably low, lest the wheeze (râle) should be heard. In short, they take every means of misrepresenting their actual condition, lest they should be deprived of food, and put upon rigid regimen. In these cases, the Professor was obliged to have repeated recourse to leeches under the clavicles, over the sternum, and other parts of the chest, wherever the wheeze could be most distinctly heard with the ear. To these means, were added blisters and severe regimen—chiefly milky and farinaceous food. If the appetite became very keen, although the pulmonary affection was not entirely dissipated, some bouillie was allowed, and, in this manner, they were kept under regimen for 10, 15, or 20 days. Nine out of these eleven patients were discharged cured, in the course of March and April, 1827. The other two remained a long time doubtful, and one appears not yet secure; the other has, ultimately, been saved from phthisis, though of a highly strumous habit, and consumptive family.

4. *Chronic Bronchitis.* In a considerable proportion of these cases, regimen alone succeeded; with the aid of some trifling narcotics. In some cases, it was necessary to employ local, and even general bleeding. By these means, all the cases recovered. The same may be said of the other chronic phlegmasiæ of the chest.

5. *Acute Gastro-Enteritis.* Of sixty cases of this disease, one proved fatal. In almost all the other cases, the disease gave way to the first, second, or third application of leeches—a few resisting the antiphlogistic treatment for a longer time. The complaint commenced with thirst, loss of appetite, general malaise, sense of heat at the epigastrium, redness of the point of the tongue, occasionally by vomiting, slight delirium, vertigo, &c. Some were taken suddenly and violently ill—others were slowly affected. Fourteen or fifteen of these cases are denominated, on the books of the hospital, "*gastric irritations*," being simple *gastro-enterites*, following a very rapid course. The symptoms of these were:—Cephalalgia, general sense of fatigue, inappetency, rednesss of the tip of the tongue, thirst, heat of epigastrium, some elevation and frequency of the pulse. Four of these cases ceded to regimen alone—the others to ten, fifteen, or twenty leeches applied to the epigastrium, seconded by rigid abstinence, and emollient mucilaginous drink. In four cases, the disease presented itself in the form of inflammatory fever, and two of them required general bleeding, in addition to the leechings. In one case, the fever was on the point of passing into the adynamic (or what is here called typhoid) state; but two applications of leeches, one of 30 and the other of 10, to the epigastrium, hypochondria, and chest, with friction of vinegar, &c. arrested the progress of the stupor, and saved the patient from a dangerous form of disease into which he was lapsing.

In two cases, there were presented the symptoms of what the Ancients denominated ileus, without knowing its cause. This was a sudden development of a circumscribed tumour in the abdomen, accompanied by vomiting and most painful colic, &c. In one case, that of a young man, aged 29 years, the tumour appeared suddenly in the night, and to ease the pain, he had swallowed a quantity of brandy and sweet oil, which were soon thrown up by vomiting. Next day, leeches were plentifully applied to the tumour, followed by fomentations. On the succeeding day, there was neither vomiting, pain, nor tumour. The bowels were opened, and, in a few days, he was discharged cured.

In the other case, the patient being a man 52 years of age, the pain was not very acute, but the vomiting was very frequent. This man had also swallowed some hot brandy and oil, which increased the sickness. The tumour was very sensible to the hand, as well as to the eye, being situated in the region of the caput coli. The pain was like that in colica pictonum, but dreadfully severe, and he begged for speedy relief from his sufferings. Twenty leeches were applied to the part. Next day, all the symptoms, and all traces of the tumour had disappeared. M. Broussais does not say much as to the real or supposed nature of these tumours in the abdomen. He thinks there is evidently acute inflammation—and possibly invagination—both of which speedily cede to the only proper mode of treatment; copious leechings and fomentations. He makes no mention of any accumulations in the colon, as the probable cause of these sudden tumours. We have seen several instances of this complaint—and one lately, in the person of a Medical Student of the Middlesex Hospital. He was, at one time, in a dangerous predicament, having neglected the complaint for a day or two. He required repeated local and general bleeding, with fomentations, calomel and opium, and smart purgation, when the disease yielded; but not before his face had assumed the Hippocratic cast, and the pulse had remained for more than 24 hours above 160 in the minute. He was judiciously treated in the beginning, by Mr. Weatherfield of Covent Garden, before we saw him.

There were some serious complications of these gastro-enterites. The most formidable was erysipelas supervening on, or succeeding, the fever occasioned by the gastro-enteric affection. Yet, even in these cases, M. Broussais did not hesitate to apply numerous leeches to the cutaneous inflammation—and, it appears, with perfect success.

The following are the principal features of the gastro-enteritis which proved fatal. The patient was in a desperate condition when he entered the hospital on the 12th November. When examined, his countenance had a very bad appearance—he was propped up in bed, breathing with much difficulty—tongue dry and red—thirst ardent—total loss of appetite—much wheezing in the right side of the chest, which seemed depressed—deafness—diarrhœa. He was bled from the arm, and leeches were applied to the epigastrium and anus—glysters were thrown up, and various means were used, but in vain. He died on the 20th of the same month. On dissection, the mucous membrane of the stomach was found of a dark brown colour, and the lower portion of ileum was studded with innumerable ulcerations. The rest of the intestines were sound; but the right lung was completely disorganized, and contained several excavations.

7. *Acute Duodenitis.* There were seven distinctly marked cases of duodenitis, and five where it accompanied other affections. In all but three of these cases, there was general jaundice. None proved fatal. In two cases, the disease appeared to be brought on by paroxysms of anger. The characteristic features were, a loaded tongue, (the crust of fur being of a grey, white, or greenish cast) bitter taste in the mouth—great diminution or total loss of appetite—a shining puffiness (renitence) in the region of the duodenum, seldom accompanied by pain. The treatment consisted in the application of 8, 10, 15, 20, or 30 leeches to the duodenal region. In the majority of cases, one application was sufficient. To this measure, warm baths were sometimes added, which helped to dissipate the jaundice. In these cases, starvation was indispensible, till the duodenitis ceased.

8. The cases of Colitis, eight in number, were easily removed by one or two applications of leeches. Diarrhœa was the characteristic feature. Rice gruel and rice pudding were found the best species of aliment.

The cases of chronic gastro-enteritis were few in number, and offered nothing particular in symptoms or treatment. Regimen is, in these cases, the mainspring of the cure.

The other diseases in the table do not require any particular notice. The following statement from the records of the Val de Grace, is certainly very favourable to the new physiological doctrine, and we entertain no doubt whatever of the superiority of the practice inculcated by this doctrine over the old routine of stimulants and tonics, in chronic diseases—“ medecine expectante” in acute.

The ratio of mortality was thus:—

| | |
|---|---|
| March, 1804 to December, 1809........ | 1 in 12 |
| January, 1810 to December, 1814........ | 1 in 10 |
| January, 1815 to December, 1819........ | 1 in 32 |
| January, 1820 to December, 1824........ | 1 in 27 |
| January, 1825 to December, 1826........ | 1 in 30. |

*Annales de la Medecine Physiologique, Mai,* 1827.

### 9. CURIOUS SPECIES OF CEREBRAL HÆMORRHAGE.

[M. Bravais. L'Hospice de Bicêtre.]

There is, says M. Bravais, a disease of the cortical substance of the brain, to which anatomists and pathologists have not directed sufficient attention. It is a hæmorrhage which generally occupies the whole of the cortical substance of the brain, giving place, first, to the formation of some small globules of blood, mixed with the nervous pulp, and afterwards producing yellowish cicatrices, extending from the external coverings of the brain down to the medullary matter. This lesion has an intimate connexion with cerebral hæmorrhage. The symptoms, at all times obscure, have not been distinguished when the disease was bounded to a small portion of brain. They are such as appertain to congestion and softening of the cerebral substance. Sanguineous effusions into the cortical structure are described by all authors; but M. Bravais has not seen any description answering to those yellow membranous-looking cicatrices which are now to be delineated.

*Case* 1. Pinard, aged 32 years, presenting, for several years, the symptoms of sombre melancholy, was observed to become rapidly emaciated during the last month of his life, and he complained much of pain in the right side of the chest, without any expectoration. He refused his food, and died on the 5th April, 1824.

*Dissection.* There was a moderate proportion of blood in the sinuses and meninges of the brain—very little serosity on the surface of the hemispheres—or in the ventricles. The arachnoid and pia mater were transparent, and easily detached from the surface of the brain. In the posterior lobe of the left hemisphere was found a crude tubercle, of small size, which readily turned out with its investing membrane. Around this tubercle, the cortical substance of the brain was reduced to a pulp, of a dark colour, in which the debris of the cerebral structure was discernible, mixed with some globules of blood. The rest of the brain preserved its usual consistence. The lungs, especially on the right side, were filled with miliary tubercles, and the bronchial glands were tuberculous.

The narrator thinks it evident, that the cerebral tubercle, and the hæmorrhagic condition of the surrounding part, came on in the latter stage of this man's existence, and had nothing to do with the melancholic affection under which he so long laboured. Of this, we do not see the evidence so very clearly. Knowing the slow growth of tubercles generally, we do not see any reasonable doubt, that the one situated in this man's brain may have contributed to the disturbance of the sensorial functions.

*Case* 2. Dupille, aged 50 years, entered the Bicêtre, on the 15th May, 1824, with the following symptoms:—Delirium, loquacity, constant motion of his limbs. The strait waistcoat was applied. *20th May.* Considerable dyspnœa—delirium—coldness of the extremities—feeble and quick pulse—puriform expectoration with his cough. He died on the 29th May.

*Dissection.* The meninges of the brain were perfectly natural, and the pia mater was easily detached from the subjacent cerebrum. There was a portion of cerebral substance, however, at the under part of the middle right lobe, which was completely impregnated, as it were, with blood, the nervous pulp and blood being intimately mixed. This was in the cortical substance:—The medullary portion underneath was quite sound. In the thorax, a pint of limpid serum was found in the right side—in the left, a pint and a half of sanguinolent watery fluid. There was not any material disease of the lungs, except in one place. The pericardium adhered at one spot, by a false membrane, to the heart. The substance of the heart was soft, and easily lacerable, especially the left ventricle, which tore in two places, merely while handling it.—REVUE MEDICALE.

It is evident that the thoracic affections, in this case, were quite sufficient to destroy life. The cerebral hæmorrhage does not appear to have produced either convulsions, or loss of sensibility or muscular power.

A considerable number of other, and nearly similar, cases are related, but we think the above are sufficient to attract the attention of pathologists to this particular lesion of the brain.

---

### 10. FATAL SICKNESS FROM PREGNANCY.

[M. Dance—Hôtel Dieu.]

Morning sickness, especially in the early stage of pregnancy, is so common as scarcely to be considered as any thing more than the effect of a natural sympathy between the uterus and stomach. Sometimes, however, is passes the usual boundary, and occasions great distress—nay, loss of life. It then becomes a morbid, instead of a natural sympathy, and we are called upon to examine into its cause and treatment.

*Case* 1. Sophy Pepin, aged 21 years, meagre, nervous, and irritable, entered the Hôtel Dieu, on the 15th April, 1826. Three months and more previously, the catamenia had stopped, and, soon afterwards, she was affected with weight and pain in the epigastrium; and considerable derangement of the general health. During the preceding two months, she was harrassed with almost constant vomiting of every thing she took, liquid or solid, attended with rapid emaciation. Yet her tongue was clean and moist, without any redness at the sides. The physician who had attended her in the city never perceived any febrile movement in the system. The epigastrium was now void of tenderness on pressure, and only a pulsation rather more than natural could be felt —sleep interrupted—habitual constipation—vomiting both night and day indifferently, preceded by a disagreeable sensation of twisting in the epigastrium. The matters ejected were often of a greenish or limpid character, and small in quantity. The patient did not think herself pregnant, and there was no enlargement of the hypogastric region.

Leeches—ice, externally and internally—and various other means had been tried, in vain, to stop the vomiting. The anti-emetic draught of Reverius was tried on the 16th, at the hospital, but ineffectually—opium plaster was applied to the pit of the stomach, with as little success. Twenty other remedies, including leeches and blisters, were put in requisition, without having the slightest effect in checking the vomiting. By the end of May, emaciation had made great progress, and now the hypogastrium began to become prominent, and pregnancy was ascertained to exist. On the 2d of June, this afflicted creature ceased to suffer.

*Dissection.* No lesion could be detected in the stomach, except a slight reddish tint in the mucous membrane. The whole of the intestinal tube was sound. The uterus rose a few inches above the pubes, and its parietes were preternaturally soft and flabby, but without any other appreciable change of structure. The membranes of the fœtus were transparent throughout; but, between these and the uterus, there were false membranes, forming a layer some lines in thickness, exactly resembling those found between the pleuræ after inflammation. The same was found between the placenta and the uterus, but more of a purulent character.

We cannot but coincide with the author in considering these inflammatory appearances in the uterus as connected with the obstinate and ultimately fatal vomiting which occurred in this case. That the affection of the stomach was purely sympathetic, is evident from the total want of all evidence of lesion in that organ after death.

*Case* 2. Aglae Leroy, aged 20 years, not married, became irregular in her menstruation in November, 1824, and soon afterwards was troubled with sickness, malaise, cephalalgia, and vomiting of bilious matters. She entered the Hôtel Dieu on the 30th December, 1824, and, at this time, she was suspected to be pregnant. The vomitings were very frequent, and there was some pain on pressure of the epigastrium, but no fever. The tongue was moist, and slightly red at the sides. She was cupped on the epigastrium, but without any benefit. Various means were employed to allay the vomiting, but they were attended with only temporary relief. In the beginning of February the sickness was as bad as ever. Her stomach would retain no kind of food, and she expired, exhausted, on the 13th of the same month.

*Dissection.* The emaciation was great—no appreciable lesion in the head or thorax—some red and softened spots near the cardiac orifice of the stomach. The uterus rose some inches above the pubis, and its parietes were exceedingly thin—scarcely a line and a half in thickness. They were also very soft, and gorged with blood. The membranes were transparent—the embryo appeared to be about three months old—there was no other appearance of disease.

The lesion in the stomach could hardly be considered as capable of producing such a catastrophe, and therefore we may fairly attribute the patient's death to the sympathetic vomiting. These two cases would, in our opinion, suggest a particular modification of treatment in the obstinate vomitings which we occasionally meet with in pregnancy—namely, repeated applications of leeches to the groins and labia pudendi. We throw out this hint for our obstetrical brethren, who have most frequent opportunities of putting the hint in practice, if they deem it proper. REPERTOIRE.

### 11. PECULIAR DISEASE OF THE HEART.

[M. Breschet. La Charité.]

Our readers will remember that, within the last year, we described three or four cases of a peculiar disease of the heart—one in the person of the late celebrated Talma—another, from our own practice, the case of the late General Kyd; and one by M. Cruveilhier. The indefatigable Breschet has dedicated a long article to this subject in the last number of the REPERTOIRE, in which he has collected nearly a dozen of instances of this very curious and rare disease. We shall endeavour to seize the more interesting particulars of this paper for the information of our readers.

1. The first case which M. Breschet has been able to find on record, is one related by Walter, the father, in the year 1759. The patient was a merchant, 50 years of age, who had, for many years before his death, complained of præcordial anxiety and palpitation of the heart. A pouch was found arising from the left ventricle.

2. After alluding to Dr. Baillie's case, the following is detailed from Zannini, published in 1816. A gondolier, at the age of 19 years, fell and bruised his chest. At the age of 25, he became affected with a complaint of the chest, attended with pain in the side, difficult breathing, cough, and bad expectoration, which symptoms were relieved by bleeding. Soon after this, he felt, for the first time, a pulsation in the *right* side of his chest, attended with a sensation as if a body was moving up and down there. He continued his avocations, and drank much wine. Two years afterwards, he became annoyed with a pain in the region of the heart, which was relieved by bleeding, but continued to return from time to time, especially on using much exertion. He had a disagreeable pulsation at the pit of the stomach. He lived two years in this condition, still pursuing his avocations, when his sufferings were a little mitigated. Having attained the age of 29 years, he suddenly expired one day, while making some corporeal exertion, after eating rather heartily.

On dissection, the lungs were found healthy. The pericardium contained a few ounces of yellow serum. From the left ventricle of the heart there went off a pouch, the size of a man's fist, which was adherent to the pericardium. This tumour opened into the cavity of the ventricle, and contained coagulated blood. The parietes of the aneurismal production varied in thickness, from three quarters of an inch to an inch and a half. There was nothing else particular in the dissection.

3. This was a negro, who was received into La Charité, on the 17th October, 1796, in the agonies of death. He died the next day, after a most profuse nasal hæmorrhage. On dissection, it was observed that the heart was of its natural size; but, from the left ventricle, there went off a tumour, nearly as large as the heart itself. The parietes of this aneurismal tumour were of a cartilaginous consistence, although they preserved the appearance of muscle. The interior of the tumour presented several albuminous layers, of considerable density, exactly resembling those seen in an arterial aneurism, except that they were more pale. The cavity of the tumour communicated, by a rather small aperture, with that of the ventricle. The aneurism appeared evidently to be formed between the muscular substance of the heart and its pericardial covering. The mitral valve was thickened and ossified. This case was recorded in the second edition of Corvisart's work. Laennec never saw an instance of the disease, and only touches on the subject, placing the disease under the head of "Dilatations partielles du Cœur."

4. Two cases of this malady were observed by M. Berard, in the dissecting room of La Charité, and whose histories were, therefore, unknown. The second of these cases offered some remarkable phenomena. The man appeared to have been about 55 years of age, and the body was very fat. On opening the chest, the pericardium presented a most enormous size. The heart was prodigiously dilated, and, at the same time, thickened in its parietes. From the summit of the left ventricle there went off an aneurismal pouch, whose interior was covered with concentric layers of organized coagulable lymph, but not of very long standing apparently.

5. The case which we gave in No. 14 of this Series, page 401, as related by M. Cruveilhier, is next introduced by M. Breschet, and this we shall, of course, pass over.

6. The next instance came under M. Breschet's own observation. On the 27th of March, a soldier, aged 49 years, was received into the clinical ward, stating that he had suffered, for six or eight months, from oppression and want of breath, especially on using exercise. The limbs and belly were now infiltrated—he was unable to lie down in bed—the left ventricle of the heart communicated a considerable impulsion to the cylinder, and each contraction was attended with a whizzing noise, (bruit de soufflet) which was very distinct, but which, however, diminished afterwards, and finally disappeared. The pulse was small, and sometimes unequal. Diuretics, purgatives, &c. were employed, with the view of removing the consecutive dropsical swellings. On the night of the 18th May, the patient suddenly became insensible, and complete hemiplegia of the right side ensued. Bleeding, leeching, &c. were used, but the hemiplegia continued, although some degree of the intellectual functions was restored. He died on the 23d May.

*Dissection.* There was serous effusion between the membranes, and in the ventricles of the brain. The right corpus striatum was of a livid colour, and completely softened almost into a fluid. This softening was exactly confined to the corpus striatum, and no other morbid appearance was seen in any part of the brain or cerebellum. The heart was nearly double its natural size, and, from the left ventricle, a small pouch went off, such as has been described. The great size of the heart was occasioned by the dilatation of the left ventricle, whose parietes, however, could hardly be said to be thickened. The tricuspid valve was in a state of induration approaching ossification. The interior of the pouch presented concentric layers of dense fibrine. The liver was granulated.

7. The case of Talma forms the next instance adduced by M. Breschet, but this has been already noticed in our own Journal.

GENERAL OBSERVATIONS AND CONCLUSIONS.

Our author remarks that the word *aneurism* has given rise to numerous altercations, discussions, and erroneous deductions. By this term have been understood various and essentially different diseases of the heart and of the arteries. It is also remarkable, that we have admitted the existence of certain morbid conditions of the heart which we have denied to the arteries, and *vice versa.* Thus, every one will allow that the chambers of the heart may become dilated, with or without *thickening*—with or without *attenuation* of their parietes; but it has been denied that there can be an aneurism of the heart produced by rupture of the fleshy parietes, and dilatation of its membranous envelopes. In respect to the arteries, it has been contended that there can be no such thing as true aneurism—that is, a general dilatation of the whole cylinder of the vessel; but that, in all cases, there is a morbid condition of the inner and middle coats, with laceration of these tissues, and the formation of a tumour on one side of the vessel, in which is contained blood, or layer after layer of fibrine. Sennertus, Fab. Hildanus, and Scarpa, maintained this doctrine. But it is too exclusive. There can be no doubt that a diseased condition of the arterial coats, with partial laceration or dilatation of them into a pouch, is the more usual form of aneurism ; but to deny that there can be a general dilatation of the calibre of the vessel, without laceration or disease of the coats, is an error. In 14 examinations of aneurismal tumours, Burns found but one in which the doctrine of Scarpa was impugned. Still this one exception in 14 cases proves that the rule is not exclusive. The present paper, and others which we have laid before our readers, evince that the heart is liable to the same kind of disease as the arteries—namely, partial attenuation, rupture, or dilatation of the parietes of some of its chambers, with the consequent formation of a pouch, containing coagulated blood, or layers of fibrine. Observation has proved, that these aneurismal pouches are almost always found to go off from the left ventricle of the heart. What can be the cause of this? M. Portal is of opinion that these pouches may form without any previous disease in the parietes of the chamber. He thinks the disease is more the consequence of violent contraction, than of dilatation of the chamber. There can be no doubt that these pouches are often seen where the rest of the parietes are in a state of hypertrophy; but M. Breschet is of opinion, and we agree with him, that, in all probability, there is an inequality of power in the different parts of the chamber, previously to the formation of these pouches. It is very curious, however, that aneurism by dilatation should be extremely common in the heart, and comparatively rare in the arteries; while, on the other hand, the aneurism by rupture or partial dilatation of the parietes is the usual form of the disease in the arteries, and comparatively rare in the heart. The reason for this difference is not very apparent. M. Breschet has attempted to lay down some symptoms as tending to shew the existence of this disease; but we consider them as quite uncertain—if not absolutely erroneous. It rarely falls to the lot of a medical man to see a specimen of this curious disease; but the one which we published—that of General Kyd, was not accompanied by any of those symptoms which M. Breschet mentions—indeed the patient never exhibited any symptoms during life, which indicated organic affection of the heart at all. Still we hold it extremely desirable that we should be acquainted with all the forms of organic disease that may be presented to our view in the course of pathological investigations; and with this view we have presented our readers with a succinct account of our learned author's paper on the above subject. The final conclusions to which M. Breschet comes are the following:—

1mo. The heart is liable to be affected by a disease hitherto undescribed by nosographers or practitioners—2dly. That the disease is chiefly found affecting the left ventricle—3dly. That the *summit* of this ventricle is the principal seat of the disease—4thly. That the tumour is aneurismal. 5thly. That this aneurism, from the mode of its formation, the state of its parietes, and the parts contained in the cyst, may be considered analogous to the " false consecutive aneurism of arteries," in the sense employed by Scarpa—6thly. That the heart is liable to the same kind of aneurism which we commonly meet with in the arteries—a circumstance that might be reasonably expected, from their structure and functions.—Repertoire.

### 12. INTESTINAL INVAGINATIONS.

[M. Buet. Hotel Dieu.]

Intestinal invaginations or intus-susceptions are often seen in young subjects who have died of other diseases, and appear to take place in the act of dying, from some convulsive or inordinate movements of the muscular fibres of the intestines. They are thus, probably, of no consequence whatever. But when a portion of small intestine is inverted into the colon—or when a portion of this last is inverted on itself, it becomes a very serious affair, and most generally fatal. It is with the view of furnishing some data for distinguishing this last and dangerous species of invagination, that M. Buet brings forward the two following cases, from the wards of Dr. Husson, in the Hotel Dieu of Paris.

*Case* 1. On the 17th of April, 1825, there was received into the above-mentioned hospital a man, aged 40 years, formerly a soldier, but now a tradesman. This man, of large stature, and strong constitution, presented the following symptoms when received into hospital:—Abdomen very hard and distended, as well as painful on pressure, especially in the *left* iliac fossa, the *right* being rather depressed, and void of pain, when pressed. The abdominal muscles appeared in strong action—pains in the loins—frequent eructations—vomiting of yellowish matters—constant inclination to stool—colicky and dragging pains in the belly—constriction about the throat. During the paroxysms of colicky pains, the left iliac region presented the figure of a large convolution of intestine, bent on itself. The poor man's countenance was indicative of great suffering—the tongue was furred—much thirst—slow pulse. The patient stated the following particulars of his illness. During ten years' sojourn at Rochefort, he had experienced several attacks of intermittent fever, which had been treated by vomits, purgatives, and tonics, &c. Afterwards he was very subject to vomiting and diarrhœa. On the 20th December, 1824, after some muscular exertion, he felt, in the *right* iliac fossa, a very acute pain, followed by colic, vomiting, and purging. These attacks came on frequently afterwards, with intervals of ease. He had occasionally discharges of blood from the bowels. The means that had been employed were leeches, fomentations, demulcent drinks, and the like. M. Husson considered the case as hopeless, and that nothing could be done but soothe the remainder of the patient's days. Thirty leeches were applied to the anus, anodyne lavements were thrown up, and fomentations were ordered to the abdomen. Little or no relief was obtained by these means. On the 23d of April, while straining at stool, the patient felt as if something had given way in his abdomen, and, in a few minutes more, he became very ill, the abdomen becoming inflated, and exquisitely tender to the touch. He died at eight o'clock the same evening.

*Dissection.* The peritoneal lining of the abdomen was every where inflamed, but there was no extravasation. Patches of recent coagulable lymph were, however, numerous. The small intestines were excessively distended with gas. Neither the cæcum nor ascending portion of colon could be found—they were carried forward, and inclosed in the last half of the transverse arch, and in the sigmoid flexure of the colon, which was as thick as a man's arm, and hard or solid to the touch. On examination, the cæcum was found lodged in the sigmoid flexure, in the iliac fossa. The invaginated portion consisted, therefore, of three parietes, as may be easily conceived, from the mode of introversion, since a portion of ileum was necessarily carried in with the cæcum and ascending arch of the colon. The invaginated parts were agglutinated together, and, in some places, gangrene had commenced. There was nothing particular in the other portions of intestine, beyond the distention, which might be naturally expected above so serious an obstruction to the evacuation of the bowels.

This case, M. Buet thinks, illustrates the effects of long-continued and drastic purgatives, to the intemperate use of which he attributes the invagination above-described. This case also shews that an intussusception of great extent may exist for a long time, and yet a passage for liquid fæces may still obtain. Up to the day before the patient's death, he had fluid evacuations from his bowels.

*Case* 2. Pradier, aged 22, who had been in the hospital at Clermont, for vomiting and diarrhœa, with colicky pains, came to Paris, in July, 1825, in a very bad state of health, and entered the Hotel Dieu, on the 8th of August, under Dr. Husson. The patient's symptoms, at this time did not portend the existence of so serious a malady as that which actually obtained. He had colicky pains and diarrhœa, with intervals of perfect ease, and his complexion was nearly natural. He was ordered emollient fomentations, diluents, and lavements. On the 12*th* and 13*th* of the same month, the symptoms were observed to be aggravated—the features were sharp—the pulse concentrated—and the iliac fossa of the left side was very painful on pressure. Leeches and opiates were prescribed. 14*th*. The pain was insupportable, and the patient felt as if something was tearing in his inside. He had nausea and vomitings, with spasmodic contractions of the fingers and other parts. 16*th*. Symptoms of peritoneal inflammation were evident, and leeches in great numbers were applied; but the disease went on unchecked, and death terminated his sufferings on the 18*th* of the same month.

*Dissection*. The peritoneum was highly injected, and there were numerous adhesions and false membranes seen in different parts, but no effusion or extravasation. Neither cæcum, ascending or transverse arch of the colon, could be found. The descending portion was as thick as a man's arm, and very hard. On performing moderate traction, it was easily discovered that the cæcum and ascending and transverse arches of the colon, together with a portion of ileum, were engulphed in the descending portion of the colon. The cæcum was found in a state of gangrene.—*Archives Générales*.

---

### 13. COMPRESSION IN ARTICULAR INFLAMMATION.

[Dr. Varlez. Military Hospital of Brussels.]

We should suppose that the Belgic surgeon has taken a hint from the writings of Dr. Balfour, in this country. However this may be, we shall give some short account of a case or two which Dr. V. has published, in the June Number of the Archives, relative to this mode of treatment.

*Case* 1. Denute, a soldier, 30 years of age, was sent into the Military Hospital of Mons, in the month of August, 1826, suspected to be labouring under some mental aberration. Shortly afterwards he was seized with violent inflammation of the stomach and lungs, which required copious depletion. When he was convalescing from this attack, he was suddenly affected with acute pain in the right wrist, succeeded by swelling of the same, and considerable constitutional disturbance. Thirty leeches were applied to the part, and fomentations to encourage the bleeding. But this brought no relief, and another 30 were applied, with poppy-head fomentations. The following day shewed a similar inflammation and tumefaction of the other wrist, without any diminution of the inflammation in the joint first attacked. Leeches were here applied, but the disease seemed to laugh Dr. V. to scorn, and pursued its course. The skin was hot—the pulse quick and irregular—the tongue dry. Dr. V. was afraid of farther sanguineous depletion, and had recourse to compression, on the principle of Velpeau in the treatment of phlegmonous erysipelas. A bandage was, therefore, applied, from the fingers to the elbow. The pain was considerably increased by this measure, and the patient begged to have the bandage removed; but the Doctor persevered, keeping the linen wet with an emollient lotion, which tightened the bandage, but did not increase the pain. The compression commenced at 10 o'clock in the morning, and, by 1 o'clock, the pain ceased. Dr. V. now applied a bandage to the other wrist, and the same augmentation of pain ensued, with complete relief in a few hours. The patient now, for the first time, fell into a refreshing sleep, of two hours duration. When he awoke, the pain had returned, but not in so violent a degree as before, and he passed a tolerable night. Next day, the constitutional symptoms had nearly disappeared. The bandages were re-applied, having become slack—the redness and swelling being considerably diminished. Next day his appetite returned. The bandages were continued for eight days, when the cure was complete. Three or four other cases, presenting similar results, are detailed by our author. We confess that we do not attach much importance to any topical measure, capable of suddenly removing the pain or swelling of articular rheumatism of the acute kind, knowing, as we do, the severe effects which so frequently succeed to rheumatic inflammation of the joints, when rudely interfered with by any local mode of treatment. This treatment by compression we believe to be one of those processes which may give a temporary eclat to the medical attendant, but may not be very profitable, in the end, to the patient. We should prefer a very moderate application of leeches to a joint in this state, followed by tepid evaporating lotions, and such internal remedies as gently open all the secretions, without violently or abruptly checking the course of the disease by any heroic remedy whatever.

We see, by this paper, that Dr. V. is about to publish a work on purulent ophthalmia, in which, he says, the surprising efficacy of the solution of chloride of lime will be demonstrated, Since he commenced using this application, he has lost no eyes; whereas, the treatment by other methods was very unsuccessful. This hint will probably be acted on by some of our surgical readers before the Doctor's work appears.

### 14. ENDERMIC MEDICATION.

[M. Martin. Hôtel Dieu.]

In spite of all the controversies which have taken place respecting the existence of a capability in the vessels of the skin to absorb substances applied to them—the general conviction, supported now by incontestible facts, is, that such absorbent power does actually exist—especially when the epidermis is removed. The method of introducing medicinal agents through this channel is by no means a mere matter of physiological curiosity. It may be turned to great advantage in many cases, where there are strong objections to the administration of medicines by the mouth. Thus, a person may become the subject of ague while labouring under chronic inflammation of the stomach or bowels—and, consequently, where bark or arsenic would be injurious. In such a case, the sulphate of quinine may be applied to the denuded surface of a blister, and the ague will be stopped, without any irritation being given to the stomach or bowels. Again, in cases of children, who have a natural repugnance to medicines, and especially to bitter medicines, the endermic medication offers a very convenient resource.

In a late Number of the Revue Medicale, the younger Martin has published several cases of intermittent fever, treated, at the Hôtel Dieu, by sulphate of quinine, applied with cerate to blistered surfaces. At first, he applied the powder over the denuded cutis, but it occasioned too much irritation, and the paroxysms were not stopped. He then mixed the quinine with cerate, and the object was obtained, without any local inflammation or pain being produced. A single case in illustration will be sufficient for our purpose.

*Case.* Leon, a soldier, of the 1st regiment of Light Infantry, had been in the hospital upwards of four months, when M. Martin took his turn of duty, on the 1st of December, 1826. He had had a pernicious remittent fever, attended with several relapses, by which he was reduced to a state of great debility and emaciation. He was also affected with dry cough, and was compelled to keep his bed, from the general state of marasmus to which he was brought by his disease. By proper means, the cough was mitigated, and the patient began to recruit a little, when, on the morning of the 20th December, he was seized with a regular fit of ague, which left the cough in a state of exasperation. The next day, being apyrectic, a blister was applied to his arm. On the third day, in the morning, the blister was removed, and the surface dressed with cerate, in which was incorporated six grains of the sulphate of quinine. That day the paroxysm was much milder, and at somewhat a later hour. The quinine was continued, six grains daily in the cerate, and fever returned no more.

We need not multiply cases, as the foregoing presents an example that will be easily imitated, where it is deemed necessary. We apprehend that this plan may be applicable to a great many morbid conditions of the system, where the internal administration of remedies would be inconvenient.

### 15. ON GYMNASTICS.

By Dr. Cassimir Broussais, Physician to the Civil and Military Gymnasium.

M. Cassimir Broussais' situation gives him good opportunities of observing the effects of gymnastic exercise on mind and body. We agree with the author, that such exercise, if properly regulated, has a powerful influence on the *morale* through the medium of the *physique*. On this account, it deserves the consideration of the statesman, the divine, the philosopher—and last, not least, the physician. It interests the latter in a double manner, as an instrument of hygiene and therapeutics. Gymnastics bid fair to exert a considerable influence on the rising generation of both sexes—the inhabitants of schools, colleges, and cities—and very particularly on soldiers, both in field and in garrison.

In this article, the author confines himself to an inquiry into the assistance furnished to the physician by gymnastics—*first*, in preserving health—*secondly*, in correcting spinal distortions—*thirdly*, in curing certain diseases—and, *fourthly*, in the acceleration, in certain cases, of the progress of convalescence.

Gymnastic exercise, by putting in action every part of the muscular system, and by diversifying the muscular movements *ad infinitum*, produces several physiological effects on the human body, which it is very necessary to know, and bear in mind. In the first place, the muscles are strengthened, and, at the same time, rendered more supple by this exercise. In the second place, these muscular movements, and their accompanying gentle succussions, accelerate the circulation, even in the most minute capillaries. Such are some of the general physical effects of gymnastics. But this agent is capable of being directed locally to this or that part—as, for example, to the dilatation of the thorax, and to the invigoration of any of the limbs, or the muscles of the trunk. By a skilful use of this measure, we may enable one organ or system to predominate over another. Thus, supposing we consult the health of a child of a lymphatic temperament, of pallid complexion, flabby flesh, and disposed to scrofula, rickets, or hydrocephalus, what is gymnastic exercise likely to effect? It will cause the muscular and sanguiferous systems to predominate over the lymphatic. In giving activity to the circulating system, it promotes the resorption of the serous fluids, and improves the complexion. In calling forth the vital action of the muscular system, and determining to the extremities, it prevents a concentration of irritation or congestion in the head, thus removing the disposition to hydrocephalus. In fine, gymnastics are capable of changing completely the original dispositions of the child into dispositions of health. Our author avers that he has seen several such instances occur at the Gymnasium. If, on the contrary, the child be neither decidedly scrofulous nor ricketty, but suffer merely from bad health; if, in fact, the child be what is commonly called "delicate;" of irritable habit, and easily fatigued, are we to treat such a patient by "rhubarb, senna, or vile purgative drug?" No; the time, says M. B. is gone by, when we should poison our patient in order to strengthen him! Let the lad engage in gymnastic exercises, gradually raised from the more gentle and preparatory, up to the more complex and difficult; and we shall soon find that these will accomplish what all the stuff in Apothecaries' Hall never could. The same holds good with the individual of bilious and melancholic temperament. Those even who enjoy good health would do well to cultivate what will give power, activity, and suppleness to their limbs, as well as confidence in themselves, and presence of mind in dangerous situations. The man of letters, too, and indeed all engaged in sedentary occupations, would find their account in resorting to gymnastics, which would most certainly lop off that endless series of ills, in the shape of piles and of fistulæ, which spring up so rankly in the field of science.

It may be asked, at what age should a child commence these exercises? Our author replies, as soon as he can walk firmly. At the Gymnasium of M. Amoros, in Paris, near the Champ de Mars, there is a class of boys from two and a half, to eight years of age, who go through the various evolutions with a remarkable degree of ease and address. It is more difficult to determine when they should be left off; for, although there is a certain period of life at which any great exertion becomes irksome, and perhaps injurious, yet it is not so easy to say, precisely, what that period is. Amongst the Greeks, old and young repaired to the Gymnasium, and we read that Galen dislocated his humerus at thirty-five.

M. Broussais proceeds to consider the effects of gymnastics in distortions and curvatures of the spine. These depend either upon caries of the vertebræ, where, of course, exercise is inadmissible, or upon partial muscular action. In a person of a weak, strumous habit, where, as M. B. believes, the osseous system is soft, and there exists a predisposition to the disease, the greater action of one set of muscles than another, induced by malposition or other accidental circumstances, is a very frequent cause of distortion. In the treatment, two indications are to be followed; 1st, the correction of deformities—2d, the strengthening of the constitution. For the first, machines have been long in use, and, with what effect, the crooked backs of nearly five out of ten of our boarding-school misses can amply tell. It is to Mr. Shaw, in this country, and M.M. Lachaise and Pravaz, on the Continent, that the merit of having combined, with the use of machines, a system of exercise for the muscles of the spine, is justly due. In France, however, they have not been content with the few ropes, and pullies, and sliding boards, employed by the late Mr. Shaw, but have sent their patients to the Gymnasium, and, as it appears, with the happiest effects. Several cases are detailed, in illustration, by M. Broussais.

*Case.* Jules B. æt. 6, of a lymphatic temperament, and puny debilitated habit, presenting a very marked projection of the spine in the dorsal region, was sent, by the advice of M. Villeneuve, to the Gymnasium of M. Amoros. On his entrance in September, 1826, there was found to be no lateral curvature, but a projection to the extent of half an inch of the spinous processes of three of the lower dorsal vertebræ. At first he evinced a great dislike to the exercises, but, after a time, this subsided; and, at the expiration of four months, there was a decided improvement in his health, whilst the prominence in the back was scarcely to be felt. At present (June, 1827) he continues to prosecute gymnastics with the happiest effects upon the *morale,* as well as the *physique.* Two other cases are related, but these our limits will not permit us to notice here. Our author thinks that gymnastics might be very advantageously combined with machines for extension, inclined planes, &c. in the treatment of these spinal distortions; in fact, at some of the institutions in Paris, for the cure of these affections, this plan seems to have been already put in practice.

The next subject noticed in this paper is—what are the complaints to the treatment of which gymnastics are applicable? Certainly not, replies our author, to acute inflammations, or active hæmorrhages—not to chronic inflammations of the viscera, to affections of the pulmonary tissue, of the heart or muscles, nor to many or most of those of the skin, &c. But in chronic gastritis and hypochondriasis, (this is a Broussaisan, remember,) in other words, in dyspepsia, gymnastics, aided by proper regimen, are an almost certain remedy. What, in fact, do we commonly recommend for these affections? Proper food—air—*exercise.* But what kind of exercise? Surely this is not to be confined to a walk or a ride. No; let the individual engage in gymnastics, not as a candidate for prizes, not with an ambition to rival the "professors," but in moderation, as a mean of health; whilst he should, at the same time, combine with them a regulated diet.

The last point which occupies the attention of our author is the efficacy of gymnastics in promoting convalescence, or rather in giving tone to the system, when this is confirmed. Caution is here required, and we must commence even with the elementary exercises, which we shall notice presently, or with the gentle use of the machines. M. Broussais observes that, if some of these were erected under the inspection of competent masters, and the immediate superintendence of the medical officers, in public institutions, particularly in the military hospitals, they might form a good mean of testing the vigour of the convalescents; in short, they might become true *dynamometers.* We have thus given an abstract of the paper of M. Broussais, and shall now beg leave, in all humility, to offer a few comments of our own. For gymnastics, in the liberal sense of the term, that is, for a system of exercise, which, without being violent or immoderate, tends to improve and develop the physical powers, we have always been the warmest advocates. We are confident, and the conviction is becoming more general every day, that exercise and a temperate diet are, after all, the true elixir vitæ. When people substitute for these a multitude of boluses and draughts, they are only poisoning themselves secundum artem, and they seem to forget altogether, that man was never intended by Nature to live upon calomel and jalap.

The gymnastics, as practised abroad, and of late introduced into this country, though, in many respects, highly valuable, are open, we fear, to several strong objections. They consist, as far as we have had an opportunity of witnessing them, in the use of a series of "bars," in vaulting, climbing, leaping, and running, together with those which are technically stiled "preliminary." These latter are principally—throwing the arms forwards, as in boxing; upwards, downwards, or backwards, as in the broad-sword exercise; jumping upon the toes; bringing the thigh to a right angle with the body, in which position it is kept for a certain length of time; and in imitating particular postures, as that of the fencer or gladiator. From this detail it will be seen, that these preliminary exercises are nothing more nor less than a species of drill, which is certainly well calculated to open the chest, give power to the muscles of the extremities, and improve the carriage. To these we think no reasonable objection can be offered, and, when once seen, they can be easily practised by most persons without the necessity for any apparatus whatever. The bars are of two kinds. A round one is placed horizontally above the level of the head, and is stiled the "horizontal pole." Upon this the principal feats are performed, some of them certainly surprising enough, both in the display of strength and agility. They, for the most part, depend upon the power of raising the head, by strength of arm, above the level of the pole, of keeping it so by means of one arm only, of bringing the knees and even toes to touch the bar, and of performing certain somersets and revolutions which would astonish the twirling dervises in the Eastern Tales. This, after all, is but a species of climbing, and as such goes to develop the chest, and enlarge the pectoral, deltoid, and biceps muscles, to a very considerable extent. The second kind of apparatus is the "parallel bars." These are two straight bars, about a couple of feet asunder, and, standing a little below the level of the arm-pit: one of which being grasped in either hand, the person lifts himself up, and walks along upon his hands, &c. Various manœuvres are gone through upon this machine also, some of them requiring a good deal of muscular force in the arm, and all of them dependent more or less upon the firmness with which the individual grasps, and supports himself upon the bars. As the former exercise was directed mainly to the biceps and flexor muscles, so this chiefly employs the triceps extensor, and latissimus dorsi. The climbing is performed upon ladders, ropes, poles, &c. but this we need not stop to describe. For the vaulting there is erected a wooden "horse," or, at least, a round log of wood placed upon legs, and so called; but here the arms and chest are still brought into play as much as, if not more than, the lower extremities; and some of the exercises we should consider dangerous, especially to a person disposed to hernia. Running and leaping complete the catalogue.

Now, on looking over the detail of these gymnastics, it is obvious that they are partial in their operation; in fact, that the chest and arms are more employed, and, by consequence, more developed than the lower extremities. That happens to a gymnast which happens to a waterman. Look at the different "stairs" by the river side, and observe this class of men. Their figures are ungraceful in the extreme. The chest is broad it is true, but the shoulders are high and square, the neck thick and short, it is "a bull-neck," and the back rounded, giving the appearance of a stoop. This, however, is not the case; for the waterman is mostly erect, the roundness of the shoulder arising from the fulness and size of the latissimus dorsi. This is the fair side of the picture; let us look a little *lower*. The nates are flattened, the thighs spare, the legs in a middle-aged waterman seldom adorned with a calf, whilst in very many, if not most, there is a degree of knock-knee, depending partly on the attenuation above and below, and partly upon weakness of the internal lateral ligament. The chest is almost Herculean, the legs miserable. And is this, after all, the form of strength? We should say not. It gives the strength requisite for the mere handling of the oar, but certainly not that adapted to a race or a wrestling match; for the man is not planted firmly on his legs. To our minds, indeed, a thorough-paced waterman presents the idea of a beer-barrel set upon spigots—so punchy above, and so lean below.

We do not mean to affirm, that gymnastics have not, in this respect, some advantages over rowing; but we do mean to say, that their operation is too partial—that the chest and arms are developed at the expense of the lower limbs, and that the figure of our modern gymnasts, so far from being graceful or beautiful, bears, in many cases, too close a resemblance to that of the waterman. We are aware that, anatomically speaking, the beauty of the male form lies in the smallness of the loins, and capaciousness of the chest; but it has been well remarked, that any peculiar or characteristic point of beauty, when carried beyond a certain extent, becomes caricature, and degenerates into deformity. Nothing can be more elegant than the fulness, and roundness of contour displayed in the pelvis and hips of a woman, and yet we apprehend there are few to be found who could admire the Hottentot Venus. We remember, some time ago, seeing at one of our theatres a rope-dancer, of the name of Antonio—Il Diavolo Antonio we think the play-bills called him. He was a short man, about five feet five, or thereabouts, and with the incessant exercise upon the slack-rope, his muscles had acquired a most extraordinary development. The gastrocnemii, the glutæi, the muscles of the thigh, and those of the chest and arms, were so prominent as to be actually visible through his tight dress at the farthest parts of the house. In the eye of the anatomist, and on the dissecting table, this would be called beauty, yet we declare that it was positively disagreeable, and even painful to look at.

We are inclined to think, that the gymnastic exercises, at present practised, are not only too partial, but too violent; and we ground this opinion on the observation of many who have engaged in them. They have become spare, and the features have assumed that rigid, pinched appearance, arising from the absorption of adeps, which we notice so universally in those who labour hard. That this is conducive to health or longevity we cannot believe; indeed, we should imagine, that if persevered in, such habits must dispose the individual to those diseases, especially of the arteries, which are so prevalent among the "hard-working" classes of the community. Be this as it may, we strongly doubt the propriety of sending young children to the Gymnasium; for if any one thing is more established in the physical education of man than another, it is this, that violent corporeal exertions at an early period of life are injurious. They most certainly tend to stop the growth, and make the individual old before his time.

Here we must conclude this article; perhaps, as it is, too diffuse. We are ready to acknowledge that there is a vast difference between criticizing a system and inventing one; and that though it is easy enough to point out a defect, it is somewhat difficult to name the remedy. All this is true; and in reply we merely say, that it is by no means our wish to abolish gymnastic exercises, though we do maintain that they should be engaged in with moderation, and that they should be combined with other means, such as dancing, walking, &c. which may counteract their too partial and injurious effects upon the economy.

---

### 16. LIGATURE OF THE SUBCLAVIAN ARTERY FOR AXILLARY ANEURISM.

By Robert Thorpe, Esq. one of the Surgeons to the Manchester Infirmary.

*Case.* Thomas Wharnby, æt. 36 years, was admitted, on Monday, April 16th, 1827, into the Manchester Infirmary, in consequence of a tumour in the axilla, which, upon examination, proved to be aneurism. About twelve months previously, he perceived a small pulsating swelling in the right axilla, the progress of which was, at first, very slow; it is now as large as a moderate-sized orange. With the exception of this aneurism, he is, in other respects, apparently healthy. The motions of the right upper extremity are not at all impeded; the only inconveniences are, a slight pain at the insertion of the deltoid muscle, and the fingers have been a little œdematous, accompanied with a tingling sensation. If pressure be made upon the subclavian artery, either above or below the clavicle, the tumour in the axilla can be nearly emptied of its contents, and its pulsations totally arrested.

Early in the month of May, I had a consultation with my colleagues, and an operation was determined upon; but the man was unwilling to have it performed, as the tumour gave him but little annoyance; and though I explained to him its real nature, the uncertainty of other plans of cure, and the bad consequences of delay in the operation, yet he wavered till the month of June, when he consented to the operation.

*June 21st.* The patient was placed upon a firm table, with his head and shoulders raised into a half sitting position. The right arm was then drawn well downwards, the face was turned towards the left side of the body, which raised the sterno-cleido-mastoideus muscle. An incision was now commenced, from the outer edge of the clavicular origin of the sterno-mastoideus muscle, and continued outwards for three inches. On dividing the platisma myoides, a small vessel sprung, and was secured, to prevent any obscurity in the further steps of the operation; and here the external jugular vein was brought into view. This was carefully held to the inner side of the wound by my assistant. Having proceeded thus far in the operation, I attempted to separate the cellular membrane (which is here very loose) with the handle of the scalpel; but, from the depth of the wound, I did not succeed to my satisfaction; nor do I suppose that the silver knife recommended by Mr. Wardrop would have answered equal to the forefingers, as one can, with them, feel the artery pulsating beneath. Having separated the cellular texture completely, the first dorsal nerve was exposed, and then from its immediate vicinity the subclavian artery itself was readily got at. The common aneurismal needle was passed underneath the artery, which was secured by a single ligature. The aneurismal sac immediately shrunk, and all pulsation was arrested. The whole operation occupied fifteen minutes. Not more than two ounces of blood were lost. The wound was dressed superficially with adhesive plaster, the aneurismal limb invested in fleecy hosiery, and the patient put to bed.

*June* 22*d*. *Four o'Clock, p.m.* Complains of oppression in his chest, accompanied with pain; also a fulness in his head, and there is a suffusion of the conjunctivæ. Twenty-four ounces of blood were abstracted, which relieved him immediately. *Eight, p.m.* Both limbs are of the same temperature, 99°.

23*d*. The blood drawn yesterday has a buffed appearance; but the patient does not complain of any pain. The thumb, he says, is most benumbed of the fingers. From this period to the 2d of July, nothing particular intervened; but on this night, at one o'clock, he *awoke suddenly*, and felt a gush of blood running down his breast. He lost about ℥xxx. which caused him to faint, and then the hæmorrhage ceased. On the 4th July, the wound was again dressed, when the ligature came away with the dressings. It is difficult to conjecture the source of the hæmorrhage on the 2d. Did the man detach the ligature by raising himself up suddenly? Yet I do not believe the blood came from the subclavian artery; *it did not come per saltum*, though it coagulated firmly. Did it come from the humeral extremity of the artery? A similar accident occurred in a case under the care of Mr. Green.

*July* 8*th*. No recurrence of hæmorrhage. The wound is granulating; the discharge is healthy, and diminishing in quantity. From this time till he left the Infirmary, which was on the 3d of September, nothing occurred worthy of remark. He was once bled to ℥xij. for a pain in the side; and occasionally small doses of sulphate of magnesia were given to him to obviate costiveness. The wound healed very progressively, though slowly; and I am much indebted to our house-surgeon, Mr. W. Guest, for his attention to the patient in preventing local sinuses, &c.

To watch the gradual return of the circulation was, in this case, exceedingly interesting. In eight hours after the operation, a tingling sensation, which had lasted from the time that the ligature was tied, now subsided; and a burning heat supervened, or, as the man expressed himself, "it felt like a fire between his shoulders and along his neck," accompanied, now and then, with a feeling, as though cold water was trickling down the arm, even to the fingers. Twenty-four hours after the operation, the arms were both of the same temperature, 99°. After a lapse of 48 hours, on making the trial, the patient could discriminate which finger was touched; yet it is rather singular that the thumb was most benumbed, though, perhaps, the most vascular. On the 6th July, I discerned a pulse in the brachial artery; but I have not been able to feel any pulsation in the radial, up to the present time, Oct. 25th, 1827. The man is now daily at work, having complete use and power of the aneurismal arm.

We congratulate British, and especially *provincial* surgery, on the adroit execution and fortunate termination, of an operation which decidedly ranks among the very foremost and most formidable to which the hand and the knife can be applied. If our readers will turn to our Number for January of last year, (1827) page 163, they will see a similar operation, by the celebrated Dupuytren, which we praised for the simplicity of the measures and the perspicuity of the narrative. The operation of Mr. Thorpe was more speedily executed, and the details are still more explicit and modest. In short, the Manchester Infirmary derives no moderate degree of honour from the dexterous and successful result of an operation, which, 30 years ago, would have been deemed equally visionary and temerarious.

---

### 17. MORBID SENSIBILITY OF HALF THE BODY.

[M. Martinet. Hôtel Dieu.]

A young man, aged 25 years, had enjoyed good health till the age of 14, when he was affected with giddiness, which returned periodically every month. On the 5th March, 1827, this young man was compelled suddenly to separate from his wife, and took the affair greatly to heart. On the evening of the same day, he was suddenly seized with a sense of weight in the right arm and leg, accompanied by a certain degree of loss of power in those parts. In this state, he fell several times while attempting to walk, and felt as if he was half intoxicated. The next day (6th), he came into the hospital, and was in precisely the condition above-described. He was bled in the evening, and sinapisms were applied to the feet. *7th.* M. Martinet himself now saw the patient. The motility of the right side was but little diminished; the principal derangement was of the sensibility. The whole of the *right half of the body* (as exactly as if a line were drawn, behind and before, from the vertex to the perineum) was exalted much in its sensibility, while the opposite side was in its normal condition. The line of demarcation between the morbid sensibility and the normal, was extremely exact. Thus, friction or stimulation on one side of the umbilicus, would excite the most insupportable feelings; while the same, on the other side, would occasion no inconvenience. A similar inequality of sensibility was observable in the two sides of the tongue and in the two nostrils; but no difference was observable in the two eyes, as to the impressions of light. The sense of smell and of taste were equal and good on both sides, though the common sensibility to touch was so unequal. Although his intellectual powers appeared unaffected, he had great difficulty, and sometimes an inability to pronounce his words, or clothe properly his ideas with language. At this he was very much vexed; and warned his medical attendants not to trust always to the words he uttered, as he was conscious of their being wrong, though he had not the power to put them right.

In other respects, this man was free from fever, slept well, and all the natural and vital functions went on regularly. *He was again bled.* *8th.* He complains of acute pain in the *left* temple; but is otherwise the same as yesterday, except that the morbid sensibility of the right side of the body is somewhat diminished. *Twelve leeches to the left temple.* *9th.* The pain is much relieved, and the sensibility of the right side still farther reduced. It was observed that the patient had made no water since the preceding day. *Catheterism—warm bath.* *10th.* The morbid sensibility was lowered to the normal degree, except in one thigh, where it was still too great. The pain of the left temple was gone, and the patient was evidently convalescent. On the *17th*, was discharged cured.

M. Martinet has headed this case "ENCEPHALITIS," and goes on to maintain that he is correct in the term. It appears that the young man, though a mechanic by occupation, was in the habit of reading a great deal of poetry, philosophy, and the BELLES LETTRES:—it is, therefore, concluded, that his ideas were habitually exalted above those in his own rank of life. After a bitter moral affliction, he is suddenly seized with weakness in the members of the right side, and a sense of intoxication. These symptoms, our author observes, must be referred to the brain. And what kind of affection was it in the brain? He can conceive no other state than that of inflammation which could produce the phenomena above-described. We are of a very different opinion. We would attribute those curious symptoms rather to *irritation* than to inflammation—and we should be much disposed to consider this irritation as a sympathetic, rather than an idiopathic affection of the brain and nervous system. We could adduce many examples of a moral affliction deranging the functions of the liver, stomach, and other digestive viscera, which deranged functions affected the brain and nervous system secondarily, and induced partial paralyses, and other disorders of the brain and spinal marrow. That there may have been some degree of vascular congestion in the *left* side of the brain, in the above case, is not improbable; but we can hardly bring ourselves to denominate the disease "*Encephalitis.*" The diminution of muscular power in the *right* side of the body might certainly be owing to some fulness in the cerebral vessels of the opposite side; but, that the *super-sensibility* of that side is to be placed to the account of *inflammation* of the brain, at a time when there was no febrile symptom present, and no apparent disturbance of the vascular system, is a proposition that might startle even Dr. Clutterbuck himself.

The case, however, is very interesting in some other respects. If we find that moral afflictions can lead to, or actually produce, an inordinate degree, or, more properly speaking, a MORBID SENSIBILITY in one half of the body, is it unreasonable to believe that the same causes may, in other instances, produce a similar morbid sensibility in certain surfaces of the interior organs, as of the stomach and upper intestines? We are convinced that these moral causes do very commonly produce this effect; but, as the sensibility of these surfaces is *organic* and not common sensibility, so the *effects* are masked, and shew themselves in a host of anomalous symptoms which are puzzling in the extreme to the medical practitioner.

---

### 18. LARYNGITIS.

[M. Martinet. Hôtel Dieu.]

In a short clinical report, published by Dr. Martinet, in a late Number of the RÉVUE MÉDICALE, an interesting case is detailed, of which we shall give the particulars in this place.

1. *Angina Suffocans.* The author avoids entering into the discussion, whether croup be a disease essentially different from cynanche laryngea et trachealis. The angina suffocans is an inflammation about the top of the air-passage, and presents some differences, according as the inflammation is seated in one or other part of the larynx or its appendages. The alteration of the voice, the sound of the cough, the distressing paroxysms of suffocation, the kind of expectoration, (when there is expectoration) suffice to characterize *cynanche laryngea and cyn. trachealis;* while, on the other hand, the tumefaction and redness of the velum palati, tonsils, and pharynx, together with the presence of crusts or exudations of different sizes, on these parts—the difficulty, or even impossibility, of swallowing—the regurgitation of liquids through the nose, these will readily mark the existence of cynanche pharyngea and tonsillaris. Œdema of the glottis may be suspected, when there is a sensation as if a foreign body were placed in the throat, and more especially when, by the finger, a kind of ring or ridge is felt surrounding the rima glottidis—attended with extreme dyspnœa. But often there is a complication of all these affections in the same subject.

In some instances, the angina suffocans does not shew unequivocal characters of *inflammation*, and then our practice is necessarily embarrassed—some trusting to depletion, some to calomel, and others to emetics and counter-irritation. In this state of vacillation as to the best method of treatment, the patient often dies by the rapidity of the disease, and the derangement which some of the most vital functions in the body experience. The following case will shew the path which we ought to pursue, where the subject is young and vigorous. It will also demonstrate the power of art over one disease at least.

*Case.* Peter Chapion, 26 years of age, of vigorous and sanguineous constitution, came into the Hôtel Dieu on the 11th March, being that day seized with acute pain in the throat, about the top of the larynx, quickly followed by difficulty of breathing, and sense of suffocation. Eight ounces of blood were taken from the arm, but the sense of suffocation continued, and made progress, without increase of frequency in the pulse, or elevation of heat on the surface. *Thirty leeches* were applied round the neck, and, the same evening, venesection was repeated to *twenty ounces.* The dyspnœa was relieved by this last bleeding, and the sense of suffocation rendered less imminent. *Mustard pediluvia.* 12*th.* The pain at the top of the throat is still acute—the voice is altered from its natural tenor, being much weaker—respiration difficult and sonorous, but not accompanied with much sense of suffocation—tumefaction of the tonsils and uvula—no appearance of albuminous exudation on any part of the fauces—the deglutition is difficult—pain on pressure of the larynx. Some of the physicians, on introducing the finger, thought they felt a swelling about the rima glottidis. There was an expuition of reddish and sanguinolent saliva—pulse easily compressible and very little increased in frequency—heat moderate—countenance indicative of anxiety—great prostration of strength—face neither pale nor flushed, but the circulation apparently impeded—inability to deviate from the upright posture. *Venesection—blister to the nucha—purgatives—sinapisms to the lower extremities.* This day passed, like the preceding, in a state of constant dyspnœa, with occasional paroxysms of sense of suffocation not, however, so threatening as to require tracheotomy. *Sixty leeches were applied round the neck*, and a cataplasm over the bites. 13*th.* The sense of suffocation is diminished, and the patient can fill the chest by a deep inspiration—expectoration very difficult, but changing in character to the muco-purulent form. *Twenty more leeches to the neck.* On examination with the stethoscope, the breathing was heard much more distinctly in one side of the chest than in the other, and the *râle muqueux* indicated inflammatory action of the mucous membrane of the bronchia. From this time, the expectoration became freer, and the dyspnœa less, until convalescence was established.

*Remarks.* The above is a good sample of that dangerous disease laryngitis. The depletion, except in the first instance, was bold and decided; but the Hôtel Dieu physicians deprived themselves of a powerful means of checking inflammatory action, by withholding nauseating doses of antimony and calomel, which increase the intestinal secretions, and save a great deal sanguineous depletion.

### 19. STRICTURE OF THE ŒSOPHAGUS.

[Hospital of Surgery.]

In the report of a fatal case of the above disease, we have noticed a strange inconsistency—if not a positive error, which the reporter will no doubt clear up.

A female was admitted, in a very weak and emaciated condition, "*with that peculiar sallow and anxious countenance which almost invariably attends* CARCINOMATOUS *disease.*"* She had, in fact, a nearly entire obstruction in the œsophagus, and the attempts to pass a bougie only aggravated her sufferings. She refused to be supported by enemata, and soon died in a state of exhaustion. On dissection, there was found a very firm contraction of the œsophagus, opposite the 10th dorsal vertebra, "produced by a thickening of the whole coats of that tube, which had, at this point, completely lost their natural texture, and had assumed the *fibro-cartilaginous* structure, which is the *sure indication of* STRUMOUS *disease.*" Below the stricture, the mucous coat of the tube was in a state of ulceration—and, at one point, the ulcer had penetrated all the coats, and communicated with the cavity of the pleura. There was nothing else remarkable in the dissection, she having, in fact, died of inanition.

We notice this case, to shew that the conductors of medical journals should be well acquainted with pathology, and the other branches of their profession, otherwise they will be at the mercy, not only of *ignorant* reporters, but of the *mistakes* of those who are *not* ignorant. We believe that a pupil of one year's study could not be found in any school of this metropolis, who would fail to blush at the assertion, that a *fibro-cartilaginous* structure was a *sure indication* of STRUMOUS disease!! The fact is, that the reporter accidentally put down *strumous* for carcinomatous; but the superintendant of the press has failed to detect so gross an error, though he boasts of being so clever as to distinguish a Greek Poem on Astronomy from a Treatise on Anatomy!

### 20. CURIOUS CASE OF HERNIA.

[Bartholomew's.]

*Case.* John Harris, æt. 51, watchmaker, was admitted into Darker's Ward, about twelve o'clock on the night of Thursday, October 4th, under the care of Mr. Earle. It appears that he has had a hernia for many years: it has caused no inconvenience; he had worn no truss; and it had not been reduced for two years. On Wednesday, while at stool, he coughed, and the tumour suddenly enlarged, and gave him considerable pain. He had been costive for two days previous to the evacuation on Wednesday. Finding the pain increase, and tenderness of the belly coming on, he sent for a surgeon, on Thursday, who tried, ineffectually, to reduce the tumour, and sent him to the hospital, at midnight of the same day. Upon examination, a large oblique scrotal hernia is found on the right side, reaching to the bottom of the scrotum, on the lower and back part of which, the testicle is to be felt very distinctly. The scrotum is tense and somewhat painful, but not much discoloured; and he complains of tenderness of the abdomen on pressure. He is

* Lancet, Oct. 27.

diligently applied by the dresser, but without effect. Mr. Earle was, consequently, sent for, and arrived between two and three o'clock in the morning. By this time the symptoms were very urgent, and Mr. Earle judged it right to have recourse to the operation without delay.

Operation.—The operation was performed about three o'clock, by Mr. Earle, who commenced by making an incision from opposite the external abdominal ring, to the lower part of the scrotum, dividing the skin and cellular membrane beneath, and exposing the superficial fascia. There was not much bleeding from the divided external pudic. A small puncture was made through the fascia superficialis, and a director introduced through it; upon which the fascia was divided by a probe-pointed bistoury. The cremaster muscle thus exposed, was next divided in a similar manner, which brought the sac into view. The superficial fascia was thicker than natural, and considerable alteration had taken place in the structure of the cremaster; it was bigger than usual, and its fibres connected into strong white tendon-like cords. The peritoneal sac was adherent to the cremaster and cellular tissue adjacent, and was next very carefully divided, and the displaced intestine brought into view, which appeared to be inflamed, but not gangrenous. The contents of the sac were evidently *large intestine*, and, on examination, found to be *the cæcum and part of the colon*, which were adherent to the sac. The fore finger of the left hand was now passed into the sac, and pushed in the direction of the external ring, through which it was readily passed, proving that no stricture existed there. Mr. Earle next ascertained that there was no cause of strangulation in the inguinal canal, or at the internal ring. The two rings were almost closely approximated. On handling the colon, Mr. Earle thought he felt something hard within it. This, he observed, was not fæces, for it was immoveable, and felt like a thickened piece of intestine within another; there being no evident cause of strangulation, but yet symptoms of it; and no small intestine to be seen entering the large gut, together with the presence of the foreign body in the colon, resembling small intestine; Mr. Earle concluded that there was introsusception. *He made an incision into the colon about an inch and a half long*, through which some dirty-coloured fluid and much flatus escaped. For what purpose this incision was made we do not exactly comprehend.* In the register of the case, written, we believe, by Mr. Earle's dresser, it is stated to have been done with a view to *lessen the bulk of the tumour*—but this must be a mistake. A little farther, however, it is said, Mr. Earle thought the urgency of the symptoms warranted it. Now we confess that we do not know on what principle cutting into the gut just *below* the introsusception was to afford relief. The coats of the divided intestine were somewhat thickened, and eversion of the villous coat took place, so as to encroach considerably on the wound. Through the wound of the

* Mr. Earle has since published the case in the Nov. Number of the Medical and Physical Journal, to which we refer.—*Ed.*

gut, and projecting into the colon, in the manner the uterus does into the vagina, might be seen a body about two inches in length, having an aperture in its middle, which appeared to be an involvulus. Mr. Earle's finger readily passed backward along the colon, through the external ring, but he was unable to push his finger in the direction towards the stomach, on account of some thickened folds of the mucous membrane.

The patient was removed from the operation table with a pallid countenance, very feeble pulse, and cold skin. He was directed to take thirty drops of laudanum immediately, in a little peppermint water, and to have a tea-spoonful of warm wine frequently administered. An injection of warm water, passed up the rectum, flowed through the artificial anus. A piece of wash leather was put upon the wound.

*Oct. 5th.*—Eight o'clock, A.M.; slight reaction has taken place, and he has not vomited since the operation; but no fæces have passed, either by the rectum or groin; some flatus is now and then extricated, previous to which he complains of griping pain in his belly. 10 o'clock, P.M. No fæces have yet passed, and the vital powers continue very much depressed; there is still tenderness of the belly on pressure; the pulse is somewhat better, and the heat greater than this morning; let him have another anodyne draught, and continue the wine.

*6th.*—During the night he has evacuated, through the artificial anus, a great quantity of feculent matter, and we find him much improved; he has very little pain; his countenance is less anxious; his pulse better, and his skin becoming warm. Let oiled silk be put so as to prevent the discharged matter from excoriating his thighs, and let him take warm broths, sago, and arrow-root, ad libitum; the sister was directed to gradually increase the dose of laudanum every night, and to give him four ounces of wine per diem.

*7th.*—He is much better this morning. His belly is soft and free from pain, and the fæces continue to be discharged at the groin. A layer of lymph has been thrown out on the surface of the peritoneal coat of the bowels, glueing them together and appearing organized. The edges of the wound are suppurating, and at the lower part of the scrotum, there is a small slough.

*8th.*—Has not altered since yesterday.

*9th.*—Fæces are profusely discharged at the groin. He complains of no pain; takes his farinaceous food and wine well; but the laudanum does not procure him sleep. The surfaces of the intestines are now covered by healthy granulations. Let the wash leather be removed, and simple dressing applied to the parts instead.

*10th.*—He is not quite so well; complains of head-ache and great depression; his tongue is furred and white, and the pulse more rapid.

*11th.*—He still passes his fæces.

*12th.*—He had a restless night; and, the sister says, has been delirious; has no pain, but great oppression and anxiety; passed a stool, through the artificial anus, to-day.

*13th.*—He is worse still; has been very restless; hiccups; the tongue is white, with a brown fur, and the temperature of his body considerably reduced; pulse quick and tremulous; let him have a pint of wine and four ounces of brandy, during the day, and fifty drops of laudanum at bed-time; he may take eggs, sago, cream tart., &c.

*14th.*—His face has a pinched and contracted appearance; he hiccups; has a brown tongue; subsultus tendinum; cold extremities, and exceedingly weak pulse; his dissolution is fast approaching; he has passed no fæces since yesterday morning; an injection of warm water was thrown up the artificial anus by an elastic gum syringe, but no fæces returned with it; give him the stimulants and laudanum, as before.

*15th.*—He has been convulsed during the night; the pulse is imperceptible, and the skin cold; no fæces have passed; he died at 7 o'clock this evening.

POST MORTEM EXAMINATION.

The body was inspected sixteen hours after death; and, on laying open the abdomen, the bowels exhibited marks of inflammation; red lines might be traced, running along their surfaces, and small patches of lymph were here and there effused; some serum, tinged with blood, had been thrown into the peritoneal cavity, but neither the lymph nor serum was abundant. It is remarkable, that, in this instance, the inflammation was not confined to that portion of the intestinal canal, above the hernia, that is, nearer the stomach than the supposed seat of strangulation; it extended along the transverse and descending arches of the colon; the cæcum, parts of the colon and ileum formed the hernia; they were matted together by lymph, which had been organized and connected into granulations. They were adherent to the sac, and the sac to the surrounding parts, particularly to the external abdominal ring.

There was no obstruction of the intestine behind or nearer the anus than the seat of the incision made by Mr. Earle; and, as we before said, this part partook equally of the inflammation. Mr. Earle expressed his doubts as to whether the part which he had considered an introsuscepted piece of ileum, was so or not. He thought that the ileo-cœcal valve might have been so elongated and diseased, as to have put on the appearance of an introsusception. If it were an introsusception, it must have been of long standing, for such perfect adhesion between the foldings of the peritoneal coats to have taken place. The finger passed, with some difficulty, through the aperture in its middle, into the ileum; the coats of which were here thickened. Behind the hernial tumour, but totally unconnected with it, and apparently arising from the cellular texture of the scrotum, was a tumour, about the size of a very large pear. In structure it appeared to consist of strong bands of ligamentous fibres, intersecting each other, with fat deposited in the interstices. It did not partake of the character of any well-known tumour, which, no doubt, caused Mr. Earle to dignify it with the designation of "rudis indigestaque moles." This tumour mainly contributed to the bulk of the scrotum. The testicle and cord were perfectly healthy, and situated behind this tumour. Mr. Earle thinks, that the tumour, pressing upon the inflamed protruded bowel, might have caused strangulation. But, as no strangulation was ever proved to exist—nay, as we have the demonstrative evidence that fæces passed without any stricture being divided, and as the inflammation was diffused over the whole extent of the intestinal canal, contrary to what is always observed in those cases where there is strangulation, we are inclined to attribute the urgent symptoms to an inflamed state of the protruded intestine which formed the hernia, and the relief which the operation afforded, to the syncope produced, which would have, as is well known, the effect of a full bleeding.—*Dissector.*

---

### 21. ANEURISM, FALSE, OF THE HEART.

[Dr. Elliotson. St. Thomas's Hospital.]

After our analysis of M. Breschet's Memoir on this curious subject *(see page 151 et seq. of this Number)* had gone to press, we were favoured by Dr. Elliotson and Mr. Alcock, with the examination of a preparation exhibiting a beautiful specimen of this rare disease.

The subject of it (James Keely, a cork-cutter) was admitted into St. Thomas's Hospital, under Dr. Elliotson, on the 2d of March, 1826. He was 32 years of age, and he complained of palpitation of the heart, especially on moving or lying down in bed—inability to lie on either side—starting from sleep—constant dyspnœa and cough, with a little mucous expectoration. He was pallid—pulse regular, but very weak. He had discharged large quantities of tape-worm by means of oil of turpentine; and he had, for many years, suffered severely from rheumatism, of which he complained at the time of his admission. He appeared to be of a remarkably quiet and placid disposition, and affirmed that his habits had always been temperate and regular.

" On applying the stethoscope, (Dr. Elliotson's note of the case) the action of the heart was found to be strong and noisy on the *right* side, both high and low down—and much less so, perhaps not more than natural, on the *left* side. I considered that the disease was in the right ventricle."

In two months, the patient was much better—had less palpitation and dyspnœa—and could lie on either side. He, also, felt stronger, and no noise could now be heard in the *right* side of the heart; but there was considerable noise in the upper part of the *left* side, and force (propulsion) in the lower part of the same. The case was very puzzling, and no determined diagnosis could be formed. The patient's dyspnœa gradually increased after this, and he died on the 15th May.

On dissection, there was seen a pouch, rising from the *left* ventricle, and extending over the right, so that the front of the latter (right ventricle) appeared to have a tumour growing on it, when the pericardium was laid open. " Would this account (says Dr. E.) for the symptoms once indicating (to me, at least) disease of the right side of the heart?"

We do not believe there are, at present, any certain diagnostic symptoms of this dangerous and fatal disease. There is reason to think, that the position of the aneurismal pouch may make a great difference in the symptoms during life. In General Kyd's case, where the pouch arose near the base of the ventricle, and presented layer after layer of condensed fibrine, no apparent derangement of the heart's function occurred till the apex of the pouch gave way and caused instant death. In general, however, the aneurism rises from the apex of the ventricle, and, in the majority of the cases on record, palpitation and dyspnœa were complained of during life.

Those who are curious to inspect a fine example of this rare disease, will have an opportunity of gratifying their curiosity by applying to Dr. Elliotson or Mr. Alcock, who, we are sure, will be happy to submit the above specimen for examination.

### 22. FATAL ŒDEMA OF THE GLOTTIS.

[Dr. Bright. Guy's Hospital.]

Leonard Evans, of remarkably stout frame, had enjoyed good health till about two years ago, when he had syphilis, which was completely cured. He had lately been subjected to sudden atmospheric vicissitudes, being a journeyman currier, in which occupation he was exposed to cold when the body was heated and perspiring. His habits had been regular and sober. Ten days before admission, he had been employed in washing skins, and got wet in his feet. The same evening, he perceived his legs swelling, and this swelling increased and spread till general anasarca was established. In this condition, he entered Guy's Hospital, on the 15th November. His urine was very scanty. Elaterium was prescribed. 18*th*. The swellings rather diminished, and a grain of elaterium was continued twice a day. 19*th*. There was a dark brown tinge in his urine, which *coagulates by heat*. Elaterium—jalap and cream of tartar. 21*st*. The anasarca a good deal reduced—makes six or eight pints of urine per diem, of a high brandy colour, which does *not* coagulate. *Dec.* 1. Complains of pain under his jaw; but the œdematous swellings are nearly gone. The urine is only four pints per diem, coagulates, and contains much blood. Bled to ℥x. This was repeated on the 2*d*, and half a grain of tartarized antimony, with two grains of opium, were given daily. 3*d*. Complains of sore throat; but he is walking about the ward, and appears much better. An ammoniated liniment to the throat. 4*th*. The throat relieved. 5*th*. He seemed much better all yesterday, and slept soundly during the night. This morning, at 7 o'clock, he suddenly complained of great difficulty of swallowing and breathing, with severe sense of constriction in his throat and chest. Fourteen ounces of blood were taken from the arm, and sixteen leeches were applied to the throat and chest. An emetic was administered. But all was unavailing, and, at 11 o'clock of the same day, he expired.

*Dissection*. No anasarca—lungs rather gorged with blood, but otherwise healthy in structure. Heart and pericardium sound. Four ounces of fluid in each side of the chest. There was nothing very remarkable in any of the viscera, except the kidneys, which presented a very curious appearance. They were large and flabby, of the darkest chocolate colour, interspersed with a few white points, and a great number of black, with a tinge of red—the whole having the appearance of polished fine-grained porphyry, or green stone. These colours pervaded the whole of the cortical substance of the kidney; but the natural striated appearance was not lost. From the left kidney, when cut through, a large quantity of blood oozed out, shewing an unusual accumulation in the organ. These appearances are represented in the fifth plate of Dr. Bright's great work.

The epiglottis was next examined, and this was found to be greatly thickened by an œdematous effusion beneath the membrane on its upper side. "It was bent into the form of a pent-house, with a sharp angle." The lower surface was also thickened, and presented a doubtful appearance of superficial ulceration. When the epiglottis was cut into, a considerable quantity of serous fluid oozed out, and the glottis itself was much contracted. Dr. B. properly observes, that there could be no doubt that the patient died of suffocation, from inflammation and consequent œdema of the glottis and epiglottis. This we believe—and surely the case was precisely one where bronchotomy would have saved the life of the patient—at least for that time. Here, then, was an instance where the physician should have explained to the surgeon the state of the case, and strongly urged the operation. If the surgeon refused, and the dissection proved the physician right in his diagnosis, what would become of the surgeon? We doubt whether he would not be amenable to the laws of his country, if prosecuted for ignorance of his profession. This case shews that the education of the surgeon should be the same as that of the physician, and, consequently, that he should be as competent to ascertain the state of an internal malady as his medical coadjutor. In fact, we conceive that the physician also should be perfectly competent to perform operations himself in cases of emergency. If Mr. Key, or any other surgeon, had been called in by Dr. Bright, and shewn the above case—and if, from punctilio, or from conceiving that he was not doing justice to his own part of the profession, by acting under a physician, he refused to perform tracheotomy—then, we say, the physician should have done it at his own risk and responsibility, rather than suffer a fellow-creature to be suffocated, This, then, is one of the evils of separating the study of the profession into distinct branches.

---

### 23. FRACTURE AND DEPRESSION OF THE FIRST DORSAL VERTEBRA.

[Guy's Hospital.]

A labourer, 30 years of age, was admitted, on the first October, under Mr. Bransby Cooper, with paralysis of the inferior half of the body. He had fallen from a ladder, with a "HOD" on his back, and against which his spine struck when he reached the ground. He was taken up insensible, and, when received into hospital, was in that state of collapse which often succeeds severe injuries. On examination, a depression was discovered about the last cervical or first dorsal vertebra, without any wound. He is now sensible, and answers questions rationally. The functions of all parts receiving nerves from the spine, *above* the injury, are undisturbed, and *vice versa*. The breathing is almost solely carried on by the diaphragm, as the chest remains dilated, in consequence of the loss of power in the abdominal muscles, while the inspiratory muscles are unparalyzed. He has feeling as low as the third rib. To be cupped (re-action having taken place) to 20 ounces—ten grains of calomel—bladder to be emptied by the catheter occasionally. *2d Oct.* Much in the same state: has had no stool—cannot void his water. To be bled from the arm—enemata—catheterism *every* 12 *hours*. *3d. In statu quo.* Blood not inflamed. No action in the bowels. *4th.* The same—bowels still inactive—enema. *5th.* Bowels cleared of a large quantity of fæcal matters—no alteration for the better. Mr. Cooper and his dressers are said to have declared several times, that there was no depression of the spinous processes. *8th.* Extensive sloughs were observed on the nates. Of this, the patient was not, of course, sensible. The patient exhibited little difference in his symptoms till the 14th October, when his urine was observed, for the first time, to be ammoniacal. On the 16th, the ammoniacal odour was much stronger—and the fatal event was evidently approaching, as the sloughs were spreading, and the various functions becoming more interrupted. He died on the 21st Oct.

*Dissection.* The subject being placed on the table, the spine was again examined. The dissector's reporter, and Dr. Hodgkin, declared there was a depression—Mr. Cooper and dressers maintained their original opinion. The membranes of the spinal marrow were more vascular than natural, and rendered tense by a quantity of serous effusion underneath. Opposite the depression, (which was found in the place above-mentioned) the membranes were lacerated, and the medulla itself completely pressed out, forming a knob, adherent to the upper portion of the thus divided spinal column. This upper portion was reddened and softened for a space of two inches from the laceration. The lower portion was not so red, but much softer—especially the posterior column, in the centre of which was a brownish disorganized matter. The body of the vertebra was fractured, and slightly displaced. The articulating processes were overlooked; but it is concluded that they were fractured. One of the lungs was inflamed and œdematous. There was nothing particular in the abdomen. The coats of the bladder were slightly thickened, and the mucous membrane, at the most depending part, "was elevated in fungoid granulations, of a dark colour; and, on their surface, small ulcers might be detected." There were also some small ulcerations near the caput gallinaginis.—Dissector.

*Remarks.* The above case is interesting in several points of view. In the *first* place, it appears to have been a case where the spinal column was completely destroyed at the part where the fracture existed; and yet the patient lived 21 days—dying, at last, as much (or probably more) from the sphacelation on the nates, as the division of the spinal marrow. In the *second* place, it is rather unusual that the ammoniacal character of the urine should not have appeared till the 14th day after such an injury to the spine, and consequent paralysis of the bladder. We suspect there was some mistake here. In the *third* place, considering that the coats of the bladder almost always sustain injury from this ammoniacal urine, should not the urine be drawn off at shorter intervals than 12 hours? Would it not be better to leave a *gum catheter* in the bladder? We know, indeed, that, in many of these cases, the urine is ammoniacal from the moment it is secreted in the kidneys; but this makes a still stronger argument for the frequent removal of the fluid, than if the change took place by mere remora in the bladder. In the *fourth* place, we think it was hardly worth the reporter's while to make so much to do about the existence or non-existence of the disputed depression in the line of the vertebræ. If the depression was so equivocal as to be denied by the surgeon and his dressers, no particular line of practice, as to an operation, was at all to be grounded on it. Where there was any suspicion of local injury, then local depletion was indicated—and this appears to have been practised. While we advocate freedom of speech and of opinion on all matters of medical science, we should be sorry to see the bad example of the LANCET's reporters followed by those of any other journal.

---

### 24. DISEASES OF THE ENCEPHALON.

[M. Raikem. Hôpital de Voltera.]

The organ of intellect—the seat of the soul—the principal centre of the nervous system—the source of sensation, reflexion, and volition—is surely a part of our corporeal fabric, the diseases of which must excite the highest interest in the mind of the medical practitioner. Medical *politics* may engross attention, for a short time, but the *practice* of our profession will prove the topic of most permanent interest in the long run. We do not, therefore, deem it necessary to make any apology for entering on the analysis of a long and important train of observations on diseases of the encephalon, chiefly drawn from the clinical wards of the HÔPITAL ST. ANTOINE, while the author was an élève interne of that institution, matured, however, by subsequent reflexion and hospital practice. The first part of this Memoir, and not the least valuable, is a minute detail of 27 cases, with the appearances on dissection; which cases form, of course, the basis of the pathological, diagnostic, and therapeutic deductions. We can only select from these cases a few of the more interesting specimens, and then proceed to an analysis of particular symptoms.

*Case* 1. A widow-woman, aged 44, was received into the HÔP. ST. ANTOINE, on the 18th October. At the beginning of the month, she became affected with erysipelas of the left leg, to which some empyrical wash was applied, and the erysipelas disappeared in three or four days. But now she was seized with fever, dyspnœa, sanguineous expectoration, and pains in both sides of the chest, which symptoms had continued six days, when she arrived in the hospital. She had then intense head-ache—flushed face—loaded tongue—thirst—pain and tenderness of the abdomen—diarrhœa—quick breathing, thoracic oppression, and frequent cough, with expectoration, containing vermillion blood. The chest sounded well posteriorly; but she could ill bear percussion on the left side and in front. Venesection to 10 ounces—blister to the original seat of the erysipelas—diluents. *7th day.* Expectoration easier, and the symptoms mitigated; but, towards mid-day, a strong exacerbation, which gained its acmé about four o'clock, when V. S. was repeated to 16 ounces, the blood issuing from the vein with astonishing impetuosity. *8th day.* Somewhat better. *9th day.* Complained of fixed pain under the ensiform cartilage, increased by coughing and by pressure. Numerous leeches. At mid-day, a violent exacerbation. In the night, the *right* side of the body became paralyzed, as to motility, but not as to sensibility—no distortion of the mouth—partial power of speech—breathing deep—cessation of the cough—pulse hard and frequent. Phlebotomy to 20 ounces. In the evening, there was some stertor—constipation—involuntary discharge of urine—difficult deglutition—pupils contracted. There is no report till the 19th day, when the organs of sense were somewhat more susceptible of impressions. She was thus bled again; but in the evening she was found comatose, and died that night.

*Dissection.* The sinuses and veins of the dura mater were nearly empty of blood, as were the cerebral veins generally—no extravasation at the base of the skull—*capillaries* of the pia mater injected. In the left hemisphere, when carefully sliced, were found several portions of brain, distinctly circumscribed, which were softened, discoloured, and, in some places, almost diffluent. In the centres of some of these portions was found a matter resembling pus, or honey. In the left ventricle was some serous effusion, and the parietes of this cavity were strongly injected. There was no other phenomenon of importance in the brain, except that the petrous portions of the temporal bone, on both sides, were found of a vivid red colour, and perforated by numerous little holes, so as to resemble the spongy part of the long bones.

In the chest, there were some marks of inflammation—some portions of lung hepatized or gorged—the mucous membrane of the bronchia of a vivid red colour, and the ultimate ramifications filled with a bloody fluid. There was nothing particular in the abdominal viscera.

*Case* 2. A young girl of 11 or 12 years of age, was received into the hospital, in the beginning of September, affected with muco-enteric fever, which, in a few days, put on a bad character, presenting the phenomena of delirium, loquacity, involuntary dejections, irregular breathing, small and unequal pulse, &c. Camphor, stimulants, blisters, &c. were employed. (This was in 1809.) About the 21st day, somnolency, passing into coma, took place, with dilatation of the pupils, paralysis of the right side, abolition of speech, involuntary stools, &c. During the whole of October, the patient continued in the following condition. She could see and hear, but appeared to receive few impressions through the medium of the other senses. She had no intellectual aberration, and manifested her wants and wishes by signs, being unable to articulate. She was disposed to somnolency, but had no coma. The pupil of the right eye was much more dilated than that of the left. There was some slight motility in the members of the right side—skin arid—pulse tumultuous and feeble—stools involuntary—rapid emaciation. This wretched state of existence was prolonged till the 6th of November.

*Dissection.* In the anterior and superior part of the left hemisphere of the brain was found a cyst, containing several ounces of sanious and purulent fluid. This cyst was from twenty to twenty-four lines in diameter, and its parietes were composed of two distinct sheets or lamina, a line in thickness, the inner one resembling a false membrane, and the outer having the appearance of a serous tissue. The surrounding cerebral substance was softened, and reduced to a pultaceous consistence. There were three or four ounces of serous fluid in that ventricle. The opposite hemisphere was sound. There was no disease in the thoracic or abdominal viscera.

*Case 3.* A female, aged 45 years, the mother of several children, had laboured, for 14 months, under excruciating pain of the whole head, exasperated, however, at irregular periods of the day and night. Various remedies had been employed, without the least effect. When admitted into the hospital, she was of a pale and yellowish complexion —all the senses undisturbed—disposition melancholy and morose—digestive organs deranged—respiration free—pulse weak—no febrile symptoms. She had some slight convulsive movements, from time to time, in her upper and lower extremities, with momentary loss of sense. She wasted, but lingered thirteen months in the hospital, still suffering from this dreadful cephalalgia.

*Dissection.* Each lateral ventricle contained two or three ounces of limpid serum. From the lateral part, on the right side of the medulla oblongata, arose a vascular kind of tumour, which spread up along the outside of the cerebellum, to which it adhered. In the centre of the right lobe of the cerebellum was a small cyst, filled with limpid serum, and having parietes composed of a thin, transparent membrane. The gall-bladder was entirely filled with calculi, and did not contain one drop of bile. There was no other disease in the body.

Twenty-four other cases are related, all shewing organic diseases of various kinds in the brain; but our limits will not permit us to analyse them. We shall, therefore, proceed to the second part of the Memoir, containing some important diagnostic observations.

Every practitioner is aware of the difficulty of ascertaining, not only the different kinds of organic disease within the cranium, but whether there be any organic disease at all. We cannot examine the brain by auscultation, as we can the viscera of the chest—nor by pressure, as we can the abdominal viscera. A solid wall of bone is placed between us and the seat of disease or disorder, and the external phenomena by which we are to be guided in our diagnosis, are exceedingly difficult to appreciate and estimate according to their real value. Although experience will not permit us to go so far as HIPPOCRATES, who says—"*Medicus sufficiens ad morbum cognoscendum sufficit etiam ad curandum*;" yet it must be allowed by all, that it is of great use to ascertain the nature of a disease, even when that knowledge convinces us that the disease is entirely beyond the reach of art. What confidence can be placed in medicine or in men, when we every day see the most fatal organic diseases pronounced as functional disorders, and their cure undertaken with the most sanguine expectations on the part of both patient and practitioner? While writing these very lines a gentleman presented himself, who was said to be labouring under *liver complaint* by one eminent physician—asthma by another—angina pectoris by a third—dyspepsia by a fourth—and hypochondriasis by several! He had carefully preserved all his prescriptions—and they would nearly make a quarto volume of curious specimens of poly-pharmacy, as our Gallic neighbours would call them. On stripping the chest, the heart was seen pulsating on the right side, and no respiratory sound could be heard in any part of the left side, except very high up towards the axilla, and at the upper part of the back, near the spine. What it was that filled up all the lower portion of the left side, we could not tell. It did not appear to be a fluid. But this we could aver, that the disease was neither hepatitis, asthma, angina pectoris, dyspepsia, nor hypochondriasis—nor did we advise the patient to swallow prescriptions for any of these complaints. It was nothing more than common honesty to tell this unfortunate man to go home—keep himself quiet—live abstemiously—attend to his bowels—and trust to GOD, since active remedies would be more likely to do harm than good. It is not likely, indeed, that he will follow our advice, for he will find many who, without taking the trouble to do more than listen to the catalogue of his symptoms, will be very ready to add a few more pages to the quarto volume of prescriptions. In this conduct we acknowledge that we did not act with worldly wisdom—for, contrary to the old maxim—honesty is *not always* the best policy—at least in these days.

But to return to the brain. We are not quite so certain as Dr. Raikem appears to be, that "*le flambeau de l'anatomie pathologique repand chaque jour la plus brillante et la plus féconde lumière sur toutes les branches de la medecine.*" The torch of the sepulchre (or, in other words, of morbid anatomy) throws every day a light on our *errors* of diagnosis—and, every day, in our opinion, reduces what have been considered as certainties, to *uncertainties!* Is this a disadvantage? No. It is better to be aware of our ignorance—than to be ignorant and think ourselves possessed of knowledge.

### CEPHALALGIA.

This is one of the most common precursory or concomitant symptoms of cephalitis—or, if you will, *softening* of the brain. But in three of the cases detailed by our author, there was no head-ache, although, in one instance, the anterior lobe of one hemisphere was a complete mass of suppuration—in another, there was a cavity in one of the lobes, with great surrounding softening—and, in a third, there were numerous unequivocal marks of intense phlogosis in various parts of the brain and its membranes. Neither is this head-ache, when it does exist, always referred to the side of the brain where the inflammation is seated. Saxonia saw a woman, who suffered from a dreadful hemecrania of the *right* side, for a long time. When she was examined after death, there was found an abscess in the *left* hemisphere of the brain. Valsalva, Morgagni, and hundreds of others, report similar observations. Our author is unable to account for these anomalies in respect to cephalalgia.

### DELIRIUM.

Is this phenomenon sometimes independent of arachnitis—and produced by inflammation of the cerebral substance itself? Our author answers in the affirmative, and states some cases and dissections, where there was violent *delirium*, but where, on examination, the brain was found inflamed and softened—the membranes unaffected. On the other hand, there are many cases on record where there was unequivocal inflammation of the arachnoid or pia mater, unaccompanied by delirium. Many cases are cited by our author from Morgagni and modern pathologists in support of this last position. These are exceptions to general rules, which it is proper to bear in mind, even in a therapeutical point of view—since we see that the absence of cephalalgia and delirium is no proof of the absence of inflammation in the brain or its envelopes.

### PARALYSIS IN THE SIDE OPPOSITE TO THAT OF THE CEREBRAL LESION.

Paralysis, and other cerebral symptoms which usually accompany inflammation or softening of the encephalon, do not always come on slowly and by degrees; but occasionally strike the patient as suddenly and violently as an apoplexy. Of this several instances are related by the author himself. Paralysis, however, in his experience, is not so frequent an attendant on encephalitis as it is generally represented to be. Of this exception, instances are given from Burserius and many modern authors, as well as from his own practice. But the wonder is, that, in numerous cases of suppuration in the brain, where cysts full of matter are found in the hemispheres, and, consequently, where there must have been inflammation preceding suppuration and softening of the brain, we shall yet find no symptom of paralysis—no disturbance of the intellectual functions. Speaking generally, however, it is when chronic inflammation has worked these changes in the encephalon, that the symptoms are obscure, or even pass unobserved. It is the same with all other organs in the body. They may be *slowly* disorganized with few of the phenomena presented by acute diseases of the same parts.

RIGIDITY OR CONTRACTION OF THE PARALYSED MEMBERS.

More than 20 years ago M. Recamier declared, in his clinical lectures, that this phenomenon depended on softening or inflammation of the encephalon. It generally does so; but this, like all other morbid phenomena, is not constant or without exceptions.

*Case.* A woman, aged about 50 years, presenting considerable embonpoint, with large head, short neck, and subject, for some time previously, to giddiness, fell suddenly down, deprived of sense and motion, and breathing stertorously. In this state she was carried to the Hôpital Saint-Antoine, in September. Her face was red and purple—eyes fixed—pupils contracted and immoveable—abolition of all motion of the *left* side, but sensation appeared to be preserved over the whole surface of the body. The left arm was paralysed, but, at the same time, *rigid.* The breathing was still stertorous, the pulse hard, full, and strong—skin burning and dry. Blood was taken from the jugular vein to the extent of 16 or 20 ounces, and sixteen leeches were applied to the neck. Lavements, &c. &c. She lingered out till the sixth day from the attack, and then expired.

*Dissection.* The vessels of the pia mater were gorged with blood. The surface of the *right* hemisphere was bulged out, and its convolutions flattened by pressure against the internal surface of the skull. In this hemisphere was found a clot of blood to the amount of two or three ounces, part of which had made its way into the ventricle of that side. The portion of brain surrounding the clot was reddened, softened, pultaceous, and lacerated in several places. The other lateral ventricle contained an ounce or two of sanguinolent serum, and there was some water at the base of the skull and in the spinal canal. The mucous membrane of the stomach was intensely red.

The above and other cases stated by our author prove that *rigidity* of the paralytic members may sometimes be seen in apoplexy—and that this rigidity is far from being constant in encephalitis, or in softening of the brain.

STATE OF THE PUPILS.

According to this author's observations the pupils, during the first period of hydrocephalus acutus, (consisting of inflammation of the membranes and sometimes of the brain itself,) are in a state of oscillation from dilatation to contraction, in consequence of inordinate sensibility; but when effusion has once taken place, the permanent dilatation and insensibility of the pupils may be generally observed. But it must be confessed, that there is the greatest uncertainty in this diagnostic symptom, and that no great dependence can be placed in the appearance of the pupils.

We must refer our readers to the third number of our cotemporary, the Repertoire, for further particulars of this lengthy investigation of M. Raikem. We have endeavoured to pick out from it those facts and observations which appeared to us most interesting in a practical point of view. The various diseased conditions of the brain and its membranes are often so obscure, and are so frequently unaccompanied by regular and appropriate symptoms, that we almost despair of their being reduced to any satisfactory system of pathology in the present state of our knowledge.

### 25. REMARKABLE DISEASE OF THE EPIPLOON.

The following melancholy case is recorded by Dr. Strambio, in an Italian journal, (*Annali di Med.*) with the appearances on dissection.

A young woman, 18 years of age, after enjoying herself at a carnival ball, began to complain of pains in the right side of the abdomen, which increased in size, and led to the suspicion that she was pregnant. Various medicines were ineffectually administered, and when M. Strambio was consulted, the patient presented the following phenomena:—face pale—pulse hard and frequent—skin burning hot—vomiting when food was taken into the stomach—abdomen large, but more so in the right than in the left side. On examination, several indolent tumours were felt, which were not painful unless strongly pressed. The left mamma was shrunk, and the right more enlarged than natural, as well as hard. There was supposed to be inflammation of the stomach, and depletion was practised, locally and generally, with fomentations, &c. It was found that a tumour pressed on the rectum, and prevented the introduction of a pipe, or the finger. All the symptoms increased in severity, and hydrothorax supervened, with infiltration of the left lower extremity. Death soon took place.

On dissection, a large effusion was found in the chest and also in the pericardium. The omentum, detached from the viscera to which it naturally adheres, was degenerated into an elastic substance, resembling brain rather than fat, occupying the whole of the abdomen, and disposed into masses of various sizes, connected by prolongations of the original omentum or membranous strips. The left kidney, the spleen, the abdominal aorta, the rectum, the ovaries, and the uterus, were involved in this morbid mass. The other organs were unaltered. The disease was considered to be caused by a chronic phlegmasia of the epiploon, excited by a fall which the young woman had experienced about a year before her death.

### 26. PURULENT OPHTHALMIA.

In our last number we mentioned that Dr. Varlez, of Brussels, had used the chloruret of oxide of calcium with great success in purulent ophthalmia. It appears by a letter from Mr. Guthrie, in the November number of the Medical and Physical Journal, that the latter gentleman has lately tested the chloruret at the Eye Infirmary, and three cases are detailed in illustration. Mr. Guthrie first employs copious venesection, and then applies the solution of the chloruret, (as made by Garden, in Oxford Street,) three times a day, by means of a brush, keeping the eyes covered afterwards with cloths wet with cold water. Purgation, with calomel, salts, &c. is also steadily employed. Mr. Guthrie seems to acknowledge the utility of this application as an auxiliary to depletion, sanguineous and intestinal.

